# Rabies Virus Targeting NIR-II Phototheranostics

**DOI:** 10.1021/jacs.5c04975

**Published:** 2025-05-02

**Authors:** Qihang Ding, Caiqian Wang, Haoran Wang, Chunbai Xiang, Zhao Wang, Yue Wang, Ling Zhao, Marc Vendrell, Jong Seung Kim

**Affiliations:** † Department of Chemistry, Korea University, Seoul 02841, Korea; ‡ National Key Laboratory of Agricultural Microbiology, Huazhong Agricultural University, Wuhan 430070, China; § Guangdong Key Laboratory of Nanomedicine, CAS-HK Joint Lab for Biomaterials Shenzhen Institutes of Advanced Technology, Chinese Academy of Sciences, Shenzhen 518055, China; ∥ Oujiang Laboratory (Zhejiang Lab for Regenerative Medicine, Vision, and Brain Health), Institute of Aging, Key Laboratory of Alzheimer’s Disease of Zhejiang Province, The Second Affiliated Hospital, Wenzhou Medical University, Wenzhou 325000, China; ⊥ Centre for Inflammation Research and IRR Chemistry Hub, Institute for Regeneration and Repair, The University of Edinburgh, Edinburgh EH16 4UU, U.K.

## Abstract

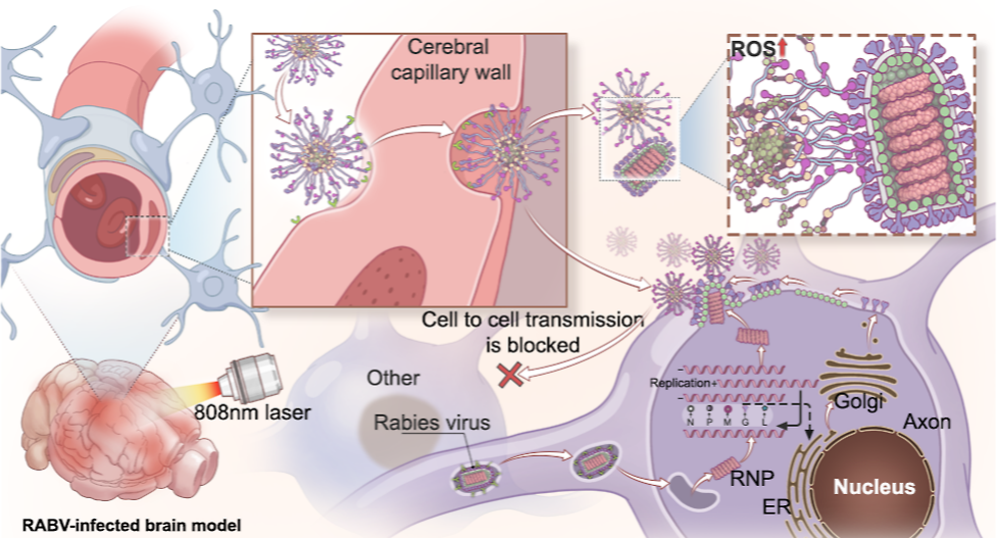

Rabies is a viral disease with an almost 100% fatality rate, primarily transmitted through bites from infected animals, with a long incubation period and no effective clinical treatments to date. Herein, we developed the first fluorescent nanotheranostic probe in the second near-infrared (NIR-II) window capable of efficiently crossing the blood–brain barrier (BBB), precisely targeting rabies virus (RABV), and enabling safe photodynamic therapy (PDT). This probe is based on a novel NIR-II organic polyacetylene fluorophore, DK, which self-assembles via a click reaction with a nanoparticle carrier, N3-PEG2000-R, that we synthesized with a high biocompatibility and BBB permeability. The probe surface is further modified with an aptamer that specifically binds to RABV glycoprotein (RVG), resulting in our final nanotheranostic probe, **DK@RA-PEG**. Upon intravenous injection into mice, it effectively crosses the BBB, localizes to the infection site, and binds to the RVG, allowing for real-time NIR-II fluorescence imaging. Additionally, it efficiently converts light energy into chemical energy without generating thermal effects, ensuring safe and effective PDT. This advanced nanotheranostic probe integrates precise targeting, deep-tissue imaging, and safe therapy, making it a promising candidate for future clinical applications in rabies treatment.

## Introduction

Rabies is an infectious disease with an almost 100% fatality rate, making it one of the deadliest infectious diseases globally.^1^ Despite being preventable, rabies remains a significant public health threat, particularly in low- and middle-income countries, where over 59,000 human deaths occur annually, predominantly affecting children.^2^ The disease is primarily transmitted through dog bites, but wild animals such as bats and foxes also serve as vectors, complicating control efforts.^3^ Effective postexposure prophylaxis and widespread vaccination programs have proven successful in preventing rabies-related deaths, yet many regions lack access to these interventions.^4^ Addressing rabies is critical not only for reducing the substantial human mortality rate but also for minimizing its economic burden on healthcare systems and advancing global health equity. Despite these efforts, however, rabies control and elimination remain formidable challenges, particularly in resource-limited settings.^5^

Photodynamic therapy (PDT) is a noninvasive treatment method that relies on the combined action of light, a photosensitizer, and oxygen.^6^ Its mechanism involves the activation of a photosensitizer by light of a specific wavelength, triggering a photochemical reaction that generates reactive oxygen species (ROS), such as singlet oxygen, which subsequently disrupts the cellular structures of targeted, diseased tissues. Initially employed in the treatment of skin cancers and other tumors, PDT has recently shown great potential in combating infectious diseases.^7^ The broad-spectrum, highly efficient antiviral action of PDT, coupled with its reduced induction of resistance, makes it a promising anti-infective therapeutic strategy.^8^ Notably, compared to photothermal therapy (PTT), PDT offers significant advantages in the treatment of rabies due to its highly localized and precise mechanism of action. By generating ROS with a diffusion range of approximately 20 nm, PDT can selectively ablate infected cells while minimizing damage to surrounding healthy tissues.^9^ This precision is particularly crucial for rabies as the rabies virus (RABV) predominantly targets the central nervous system (CNS), where any nonspecific damage can result in severe neurological consequences. In contrast, PTT relies on the generation of heat, which can spread to adjacent healthy tissues, posing a higher risk of collateral damage, especially in a sensitive neural environment. By producing localized ROS, PDT constitutes a safe therapeutic strategy with potential for combating rabies infections within the nervous system.^10^

The second near-infrared (NIR-II) window (1000–1700 nm) imaging-guided PDT represents an emerging and advanced technology that combines high-resolution imaging with PDT.^11^ NIR-II imaging offers optimal tissue penetration and accurate imaging of deep lesions with high signal-to-noise ratios.^12^ Additionally, the low scattering properties of NIR-II light ensure efficient light energy delivery, enhancing the therapeutic efficacy of PDT, especially in the elimination of viruses in deeper tissues.^13^ Therefore, NIR-II imaging-guided PDT enables viral suppression within neural regions and holds promise as a novel therapeutic strategy for the treatment of rabies after the onset of clinical symptoms.

The delivery of photosensitizers to infection sites within the CNS, particularly in rabies-infected regions, remains challenging. The blood–brain barrier (BBB) prevents most drugs from entering the brain, thus impeding the delivery of therapeutic agents for the treatment of rabies. Drug delivery systems like liposomes and nanoparticles can be used to improve bioavailability but often exhibit uneven or unpredictable distribution and degradation *in vivo*.^14^ The delivery of drugs to the CNS also often requires high dosages, which increases the risk of side effects and potential toxicity. In conclusion, the crossing of the BBB, uneven drug distribution, and efficacy of virus elimination have complicated the design of therapeutic interventions for rabies, especially after RABV has invaded the CNS.^15^ This unmet therapeutic need has driven the development of this work.

**Figure 1:**
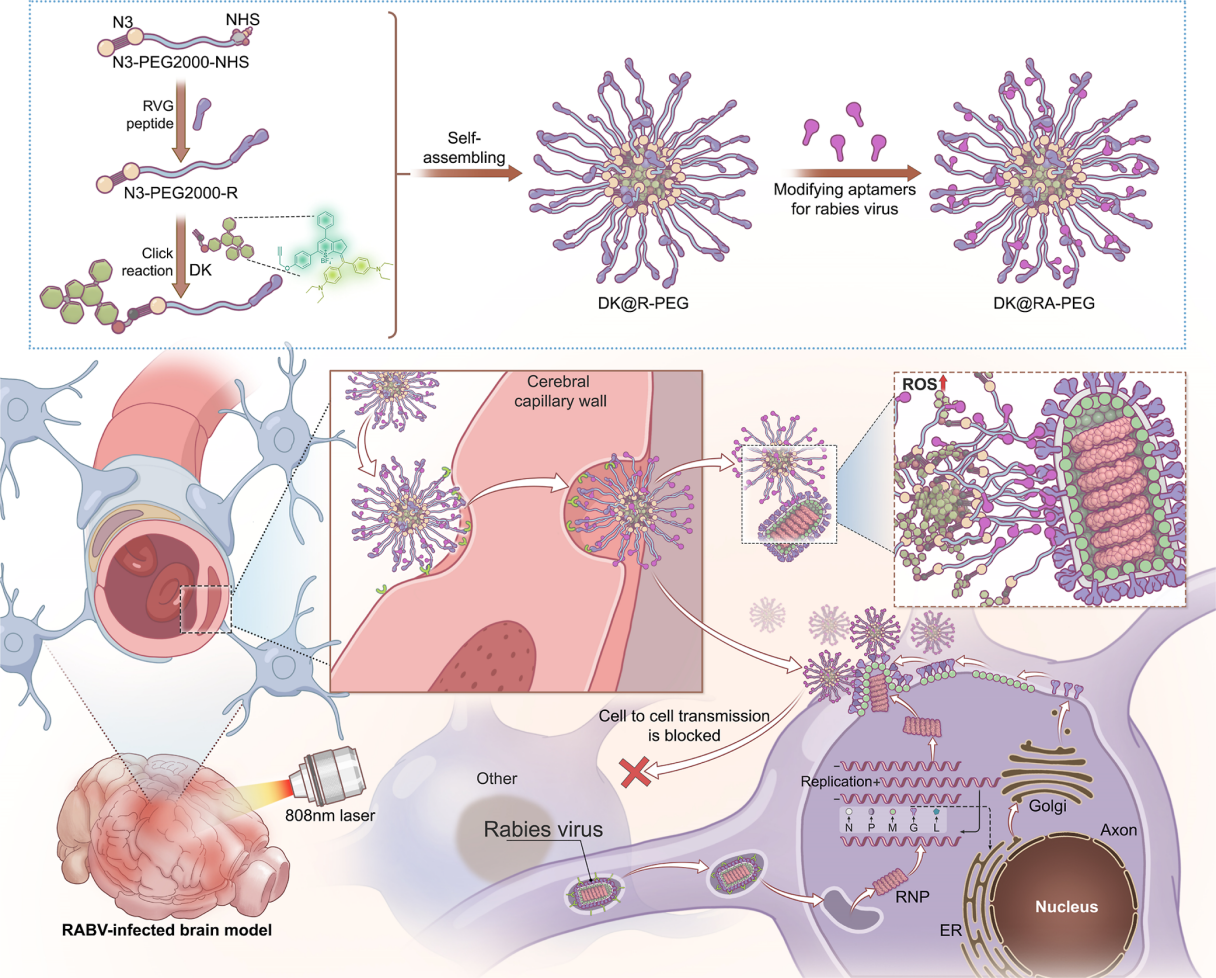
Schematic diagram of the construction of **DK@RA-PEG** and its precise targeting of RABV through the blood–brain barrier for photodynamic therapy.

Herein, we developed the first NIR-II fluorescent nanoprobes to target *in vivo* RVG, the key viral component infecting the CNS, after BBB crossing. These nanoprobes enable safe NIR-II imaging-guided PDT for both the detection and treatment of rabies. Our nanoprobes are based on a novel NIR-II organic polyacetylene fluorophore (DK) that emits at a maximum wavelength of 1000 nm, with a tail extending into the NIR-IIa (1300–1400 nm) region. DK exhibits a large Stokes shift (235 nm), high quantum yields, exceptional photostability, and selective production of ROS without thermal effects. Our nanoprobes combine amphiphilic and biocompatible polymers with a BBB-crossing and RABV-targeting peptide. This resulted in a highly biocompatible nanocarrier, N3-PEG2000-R, with excellent BBB penetration capabilities. Next, we utilized click chemistry to link the azide group on one end of N3-PEG2000-R to the alkyne group on DK, forming the nanoprobe DK@R-PEG. Finally, we functionalized the nanoprobe with DNA aptamers that specifically target RVG, resulting in the final nanoprobe, **DK@RA-PEG**. Both in vitro and in vivo experiments demonstrated the NIR-II imaging capabilities of **DK@RA-PEG** along with its effectiveness in inactivating RABV. Specifically, the survival rate of rabies-infected mice treated with **DK@RA-PEG** was significantly improved (∼40%) compared to the control group. Behavioral assessments indicated no abnormalities in the treated mice, further validating the excellent safety profile of our photodynamic therapy. Overall, our results indicate that irradiated **DK@RA-PEG** can serve as a first-in-class chemical strategy for the PDT treatment of rabies (Figure 1).

## Results and Discussion

### Molecular Design, Synthesis, and Characterization of Phototheranostic Probes

**Figure 2:**
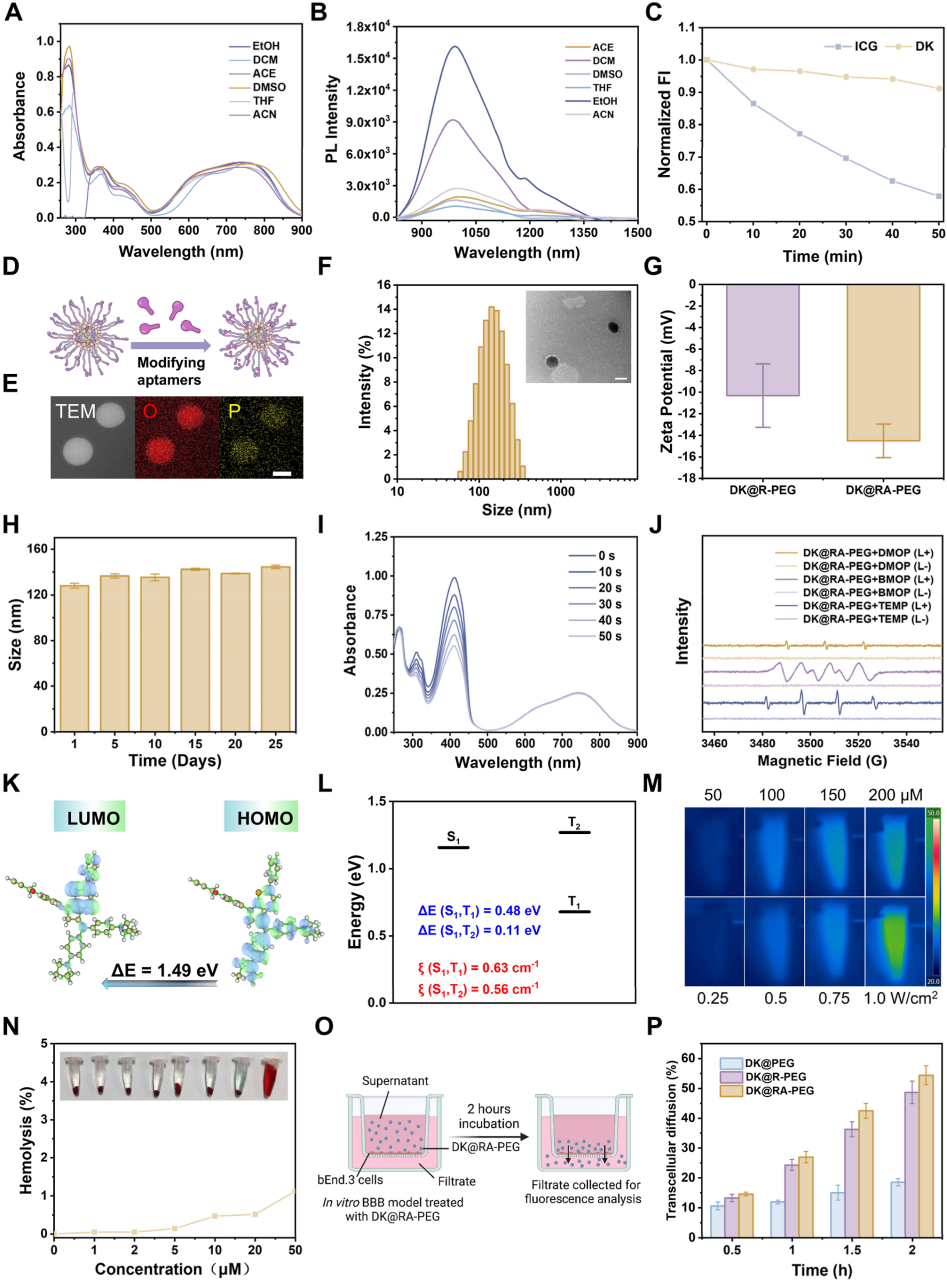
Nanoplatform construction and characterization. (A) Absorbance and (B) fluorescent emission of DK in 50 μM different solvents. (C) Photostability of DK and ICG in phosphate-buffered saline (PBS) buffer under continuous 808 nm irradiation (1.0 W cm^–2^) and fluorescence intensity (FI ) were recorded at predetermined time points (0, 10, 20, 30, 40, and 50 min). (D) Mechanism diagram of DK@R-PEG-modified aptamers; (E) elemental mapping of **DK@RA-PEG** (P and O elements) (scale bar = 100 nm). (F) DLS size distribution and the representative TEM image of the **DK@RA-PEG**. Scale bar: 100 nm. (G) Zeta potential of D@RA-PEG and **DK@RA-PEG** (*n* = 3). (H) The size stability of **DK@RA-PEG** (20 μM) in DMEM for 25 days. (*n* = 3) (I) Absorption spectrum of DPBF solution containing 20 μM **DK@RA-PEG** under 808 nm irradiation (0.5 W/cm^2^) (J) ESR signals of DMPO, BMPO, and TEMP (100 mM, respectively) in the presence of **DK@RA-PEG** (50 μM in deionized water) before and after 808 nm laser irradiation for 5 min (0.5 W/cm^2^) (K) DFT calculation of frontier molecular orbitals and energy gap energies for DK. (L) Calculated lowest excited singlet (S_1_) and triplet energy (T_1_ and T_2_) levels of DK (CAM-B3LYP/6–31G (d) level) and calculated spin–orbit coupling (SOC) constants between the lowest excitation singlet (S_1_) and triplet energy level (T_1_–T_2_) of the DK. (M) IR thermal photos of **DK@RA-PEG** (50, 100, 150, and 200 μg/mL) under the laser irradiation (808 nm, 0.5 W/cm^2^) at various concentrations and **DK@RA-PEG** (100 μg/mL) under the various 808 nm laser power irradiations (0.25, 0.5, 0.75, and 1 W/cm^2^). (N) Hemolysis rate of **DK@RA-PEG** at different concentrations. (O) Schematic representation of experimental models to study the BBB crossing efficiency of DK@PEG, DK@R-PEG, and **DK@RA-PEG** in vitro. (P) Statistical results of the mean FI of DK@PEG, DK@R-PEG, and **DK@RA-PEG** in the lower chamber. (*n* = 3).

Following the synthetic methods previously reported by our groups,^16^ we designed and synthesized DK as a novel NIR-II small organic photosensitizer. The molecule was comprehensively characterized using ^1^H NMR, ^13^C NMR, and ESI–HRMS (Figures S1–S3). DK exhibits excellent optical properties, displaying broad absorption in various common organic solvents with a maximum absorption wavelength of 755 nm and an emission wavelength of approximately 990 nm. The fluorescence quantum yield was determined as 0.11%^17^ (Figures 2A,B and S4). Moreover, the absorbance and fluorescence emission intensities increased linearly with DK concentration (Figures S5 and S6), indicating no aggregation under these conditions. The scattering and penetration capabilities of DK were further investigated using 1% lipid emulsion as a tissue-mimicking phantom. DK exhibited a penetration depth of approximately 8 mm, surpassing that of indocyanine green (ICG), the only FDA-approved NIR clinical imaging agent to date (Figure S7). Furthermore, DK displayed superior photostability compared with ICG. Under continuous irradiation for 50 min, the fluorescence emission of DK showed negligible attenuation, whereas ICG experienced a significant decrease (Figure 2C), highlighting the notable photostability of DK.

The BBB is a critical barrier to maintaining CNS homeostasis and inherently limits drug delivery. To enhance the water solubility, biocompatibility, and BBB permeability of DK, we conjugated the photosensitizer to the amphiphilic polymer N3-PEG2000-NHS with the RVG peptide (sequence: YTIWMPENPRPGTPCDIFTNSRGKRASNG), which is known for its advantages in neural-targeted delivery systems.^18^ The resulting peptide-modified polymer (N3-PEG2000-R) was structurally characterized via mass spectrometry and HPLC (Figures S8–S10). Subsequently, DK was conjugated to the azide group on the peptide-modified polymer through a click reaction, forming self-assembled nanoparticles DK@R-PEG.

Aptamers are short, single-stranded DNA or RNA molecules with exceptional target-binding capabilities. To further enhance the targeting ability of DK@R-PEG toward RVG, we functionalized it with an aptamer previously developed by our group via SELEX.^19^ This aptamer specifically recognizes RVG (aptamer sequence: 5′-TCACTCCACTAATCAACAATTCATTTATTACAATCGCTGG-3′, amine-modified at the 5′ end) (Figure 2D). The synthesized nanoplatform **DK@RA-PEG** was characterized by scanning transmission electron microscopy–energy-dispersive X-ray spectroscopy (STEM–EDS) elemental mapping, revealing the presence of phosphorus in **DK@RA-PEG**, while no phosphorus signal was detected in DK@R-PEG (Figures 2E and S11). Furthermore, X-ray photoelectron spectroscopy (XPS) analysis confirmed the presence of a P 2p characteristic peak in **DK@RA-PEG** (Figure S12). High-resolution XPS spectra further validated that the binding energy of P 2p falls within the typical range of phosphate groups, demonstrating the successful conjugation of the aptamer onto the **DK@RA-PEG** surface (Figure S13). Additionally, energy-dispersive X-ray spectroscopy (EDS) analysis also confirmed the presence of phosphorus in **DK@RA-PEG**, further supporting the effectiveness of aptamer modification (Figure S14). Collectively, the results from STEM–EDS elemental mapping, XPS, and EDS mutually corroborate the successful chemical functionalization of **DK@RA-PEG**. Moreover, quantification based on the fluorescence signal of Cy3-labeled aptamers revealed a modification efficiency of 47% for the aptamers on **DK@R-PEG** (Figure S15).

The optical properties of **DK@RA-PEG** were then characterized in deionized water. As shown in Figures S16 and S17, **DK@RA-PEG** exhibited absorption and emission peaks at ∼747 and ∼917 nm (808 nm excitation), respectively, similar to those of DK. Additionally, assessments of scattering and penetration using lipid emulsion phantoms and photostability under continuous irradiation indicated that the peptide and aptamer modifications did not alter the photophysical properties of DK (Figures S18 and S19). Moreover, dynamic light scattering (DLS) and transmission electron microscopy (TEM) revealed uniform spherical distributions with average sizes of approximately 174 and 157 nm for **DK@RA-PEG** and DK@R-PEG, respectively (Figures 2F and S20). The slight size increase in **DK@RA-PEG** is attributable to the aptamer’s molecular volume. Zeta potential measurements showed that **DK@RA-PEG** (−14.5 mV) exhibited a higher negative charge than DK@R-PEG (−10.32 mV), likely due to the inherent negative charge of the aptamer (Figure 2G). The enhanced negative surface charge reduces the chance for recognition and clearance by the mononuclear phagocyte system,^19^ thereby prolonging circulation time and improving target tissue distribution. Stability studies confirmed that the hydrodynamic sizes of DK@R-PEG and **DK@RA-PEG** remained unchanged for 25 days in cell culture media, underscoring the excellent stability of this platform (Figures 2H and S21).

Subsequently, the photosensitizing properties of DK and **DK@RA-PEG** were assessed using 1,3-diphenylisobenzofuran (DPBF) as a reactive oxygen species (ROS) probe. Under 808 nm laser irradiation (0.5 W/cm^2^), the absorbance at 410 nm decreased significantly for DK and **DK@RA-PEG** solutions containing DPBF, whereas the absorbance of DPBF alone remained unchanged, indicating their PDT potential (Figures 2I, S22, and S23). The ROS generation efficiency of DK, quantified using ICG as a reference, was determined as 0.172^20^ (Figure S24). Furthermore, electron paramagnetic resonance (EPR) spectroscopy employing BMPO, DMPO, and TMEP verified that **DK@RA-PEG** produced multiple ROS species, including ^1^O_2_, superoxide anions (O_2_^•–^), and hydroxyl radicals (^•^OH), under 808 nm laser irradiation (0.5 W/cm^2^), thereby demonstrating its capability to induce both type I and type II PDT (Figure 2J).

To gain deeper insight into the photodynamic properties of DK, we conducted density functional theory (DFT) and time-dependent DFT (TD-DFT) calculations.^21^ At the B3LYP/6-31G(d) level, electronic structure analysis revealed distinct frontier molecular orbital distributions: the highest occupied molecular orbital (HOMO, −7.04 eV) was predominantly localized on the electron-rich methanofullerene unit, while the lowest unoccupied molecular orbital (LUMO, −5.55 eV) resided on the electron-deficient thiochroman moiety (Figure 2K). This spatial separation reduces recombination energy, thereby narrowing the bandgap to 1.49 eV—an indicator of intramolecular charge transfer (ICT). The small singlet–triplet energy gap (Δ*E*_ST_) facilitated efficient intersystem crossing. To further elucidate the underlying mechanism, TD-DFT calculations were performed at the PBE0/def2-TZVP level, revealing a minimal energy difference between the S_1_ and T_2_ states (0.11 eV). Moreover, the substantial spin–orbit coupling (SOC, 0.56 cm^–1^) further promoted triplet-state formation, corroborating the strong ROS generation potential observed experimentally (Figure 2L). Furthermore, infrared thermography demonstrated that **DK@RA-PEG** exhibited negligible photothermal conversion under 808 nm laser irradiation. Even at a laser power of 1 W/cm^2^, the temperature increased to only 31.9 °C, highlighting its low photothermal activity (Figures 2M, S25, andS26). The photothermal effect of DK@RA-PEG was nearly abolished, likely due to the restricted vibrational and rotational freedom of 4,4′-Bis(diethylamino)benzophenone in the aggregated state.^7^ The lack of photothermal effects minimizes potential heat-induced damage to surrounding tissues, making this platform a suitable approach for rabies treatment in the CNS. The hemolysis tests demonstrated minimal levels of hemolytic activity, with hemolysis rates of approximately 1%, thereby demonstrating excellent biocompatibility (Figure 2N).

Finally, we evaluated the BBB permeability of **DK@RA-PEG** using an in vitro bEnd.3 cell model. Transwell assays demonstrated significantly enhanced translocation efficiency for **DK@RA-PEG** and DK@R-PEG compared to that for DK@PEG (Figure S27). Interestingly, the APT modification appears to enhance the BBB permeability of **DK@RA-PEG**. Based on previous studies, we speculate that APT modification may improve RVG peptide-mediated transcytosis by strengthening electrostatic interactions and leveraging endogenous transport pathways, thereby increasing BBB permeability (Figure 2O,P).^22^ This result underscores notable penetration capabilities, highlighting the potential for BBB crossing and advanced therapeutic applications.

### **DK@RA-PEG** Exerts Potent *In Vitro* Antiviral Effects against RABV

**Figure 3:**
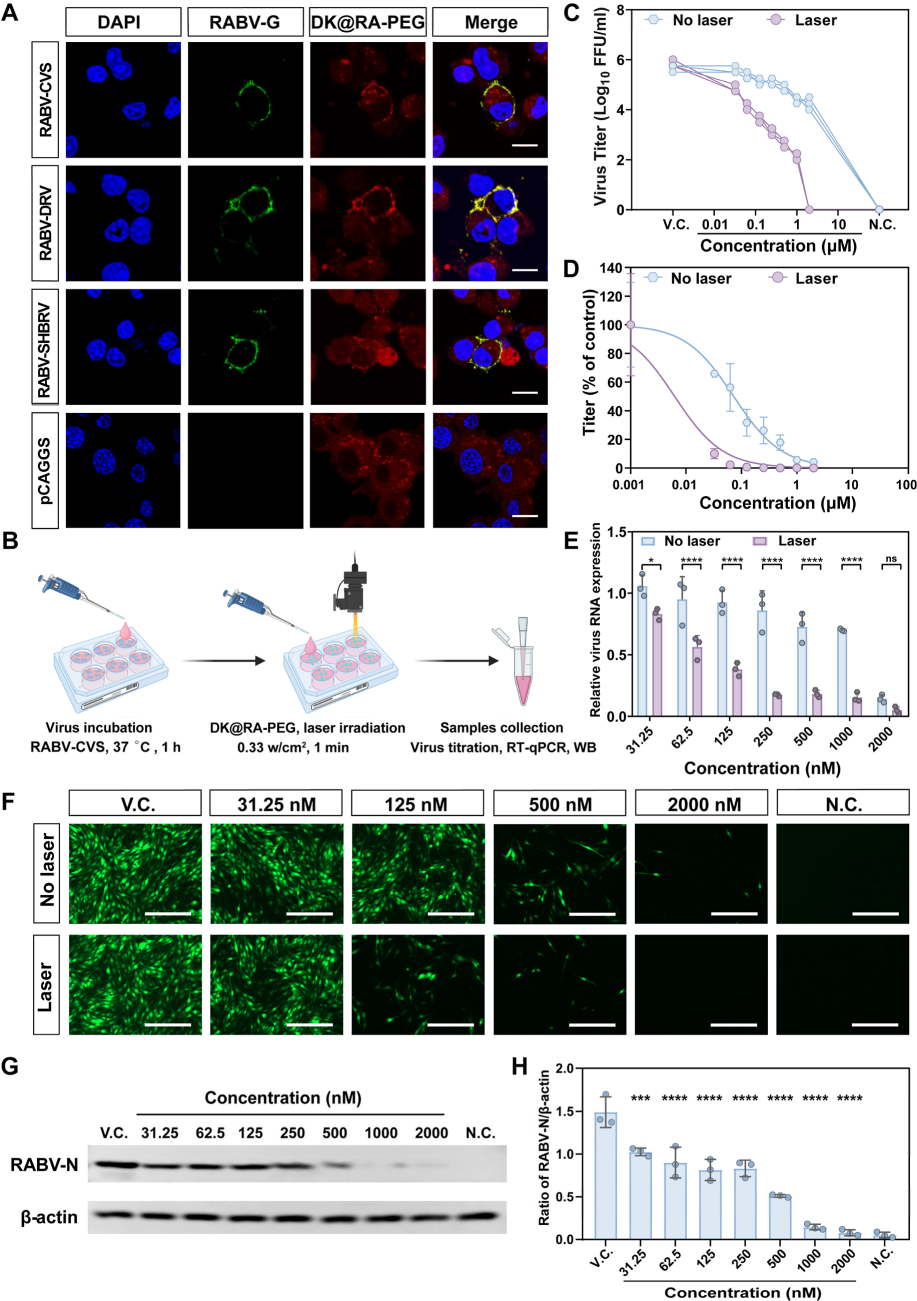
Antiviral activity of **DK@RA-PEG** against RABV *in vitro*. (A) The targeting ability of **DK@RA-PEG***in vitro*. N2a cells were transfected with plasmids encoding glycoproteins of different RABV strains (RABV-CVS, RABV-DRV, and RABV-SHBRV), and blank pCAGGS plasmid was used as a negative control. After 24 h, cells were incubated with **DK@RA-PEG** for 12 h and then incubated with RVG-specific mAb, Alexa Fluor 488 goat anti-mouse IgG, and DAPI. Scale bar, 10 μm. (B) The general procedure for *in vitro* photodynamic inactivation of RABV. (C) Virus load in supernatants was determined by virus titration (*n* = 3). (D) The 50% effect concentration (EC_50_) index of DK@RA relative to RABV-CVS. (E) RT-qPCR analysis of RNA expression of RABV-CVS (*n* = 3). (F) Immunofluorescence assays of RABV-CVS-infected BSR cells treated with different concentrations of **DK@RA-PEG** under 808 nm laser irradiation (0.5 W/cm^2^) for 1 min or not, scale bar: 200 μm. (G) Representative Western blot image of RABV-CVS-infected BSR cells treated with different concentrations (31.25 nM to 2000 nM) of **DK@RA-PEG** with 808 nm laser irradiation (0.5 W/cm^2^) for 1 min. (H) The mean gray values of the RABV nucleoprotein (RABV-N) levels in BSR cells were analyzed by Western blot using ImageJ (*n* = 3). N.C., negative control; V.C., virus control. Data in (C), (D), (E), and (H) are represented as the mean ± SD. Statistical significance was calculated by two-way ANOVA with Tukey’s multiple comparisons test in (E) and one-way ANOVA with Tukey’s multiple comparisons test in (H). *P < 0.05, ***P < 0.001, ****P < 0.0001, ns, not significant.

To assess the cytotoxicity of **DK@RA-PEG** in cells, we treated BSR and N2a cells with different concentrations of **DK@RA-PEG** followed by irradiation with the NIR laser (808 nm, 0.5 W/cm^2^) for 1 min. Cell viabilities were measured by the Cell Counting Kit-8 (CCK-8) assays, and we observed no significant differences in viability between the **DK@RA-PEG**-treated groups and the mock control group (Figure S28A,B), indicating that PDT on its own has no toxic effects on the cells used for modeling infection. Moreover, the cytotoxicity of **DK@RA-PEG** was assessed in the brain microvascular endothelial cell line Bend.3, following a 24 h coincubation with different concentrations of the compound. The CCK-8 assay results revealed minimal cytotoxic effects (<10% reduction in viability at 2000 nM), indicating favorable biocompatibility for potential neurovascular applications (Figure S29). RVG, a crucial protein facilitating the entry of RABV into host cells, was overexpressed in N2a cells with different RABV strains (CVS, SHBRV, and DRV) to assess the targeting capability of **DK@RA-PEG**. Immunofluorescence analysis demonstrated colocalization of **DK@RA-PEG** with RVG (Figure 3A), confirming that the aptamer-modified **DK@RA-PEG** successfully targets RVG-expressing cells. Given the excellent photophysical properties and targeting ability of **DK@RA-PEG**, the photodynamic inactivation potential of it against RABV *in vitro* was then investigated. As shown in Figure 3B, BSR cells were infected with RABV-CVS at a multiplicity of infection (MOI) of 0.01 for 1 h at 37 °C. Then, the supernatant was removed, and cells were further incubated with fresh medium containing **DK@RA-PEG** and irradiated with the NIR laser (808 nm, 0.5 W/cm^2^) for 1 min. A control group without laser irradiation was also performed. After 36 h of incubation, the supernatant and cell lysates were collected for further analysis. The result of the virus titer showed that **DK@RA-PEG** effectively inhibited RABV-CVS replication in BSR cells, even without laser irradiation (Figure 3C,D). However, the effect was even more obvious in the laser irradiation group, where inhibition was 12 times stronger (EC_50_ = 6.23 nM) than in the no-laser group in BSR cells (EC_50_ = 72.31 nM, Figure 3C,D). Similar results were observed by RT-qPCR, where the levels of viral RNA in BSR cells decreased significantly upon treatment with **DK@RA-PEG** (Figure 3E). Moreover, immunofluorescence and Western blotting assays also confirmed lower levels of viral protein in the infected cells. The fluorescence foci and the expression of the RABV nucleoprotein in the BSR cells both decreased when the concentration of **DK@RA-PEG** increased from 31.25 to 2000 nM under 808 nm laser irradiation (Figure 3F–H). Additionally, **DK@RA-PEG** also showed potent inhibitory effects against two other RABV strains, SHBRV and DRV (Figure S28C–E). In summary, our results indicate that 808 nm laser irradiation of **DK@RA-PEG** can effectively inactivate various RABV strains *in vitro* and prevent host cell infection to inhibit the spread of RABV.

**Figure 4:**
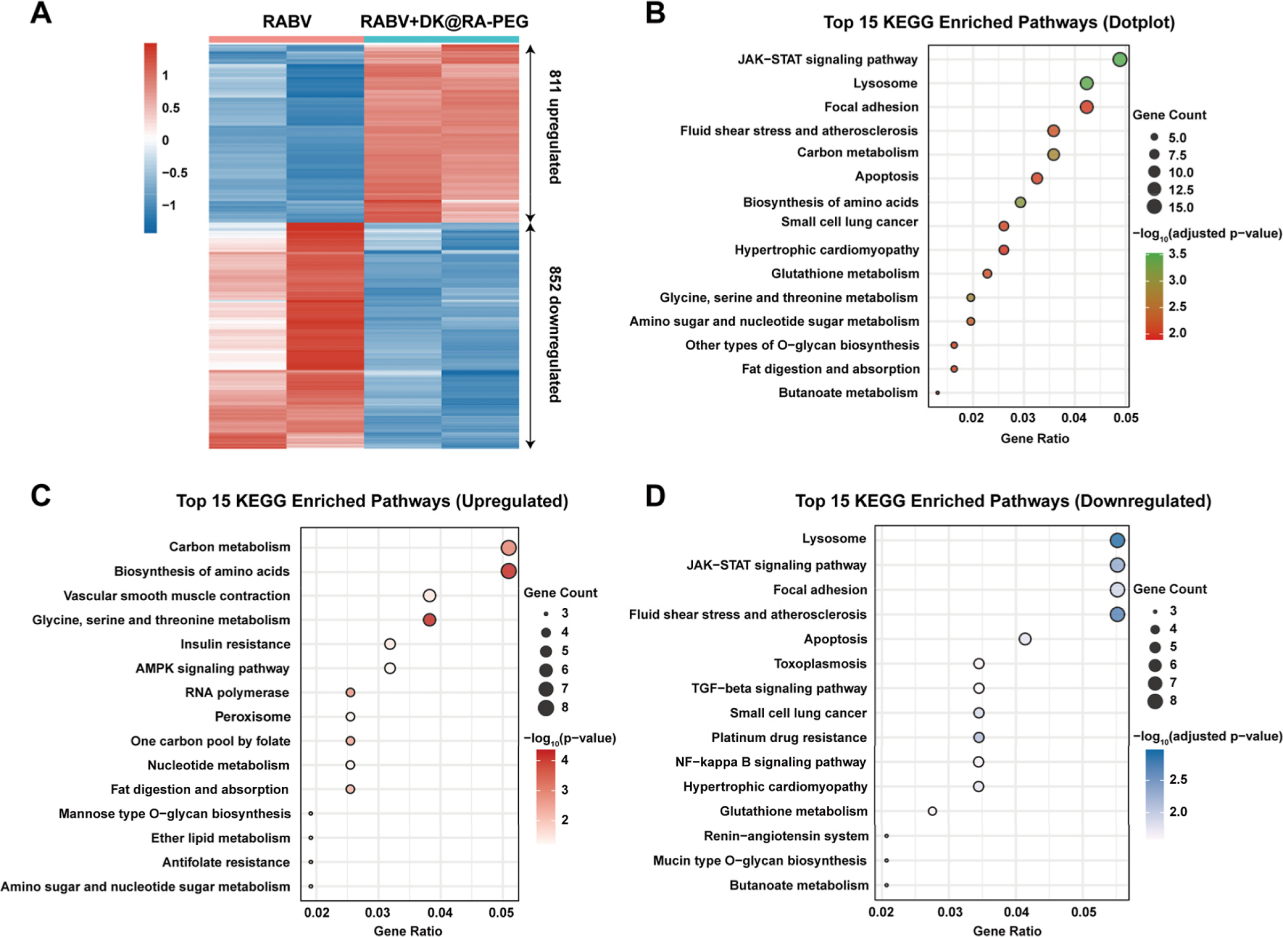
Transcriptome analysis of BSR cells treated with RABV and **DK@RA-PEG** + NIR laser. BSR cells were infected with RABV (CVS–B2c) for 1 h at 37 °C and then treated with **DK@RA-PEG** and irradiated with the NIR laser (808 nm, 0.5 W/cm^2^) for 1 min. Cells were collected 36 h after infection for RNA sequencing. (A) Heatmap showing differentially expressed genes between the **DK@RA-PEG** and NIR laser-treated group and the untreated control group (Control), as determined by RNA-seq (adjusted *P*-value < 0.05 and |Log_2_ Fold Change| > 0.5). (B) Top 15 enriched Kyoto Encyclopedia of Genes and Genomes (KEGG) pathways associated with differentially expressed genes following **DK@RA-PEG** treatment and NIR laser irradiation. Pathways are ranked by −log_10_(adjusted *P*-value). (C,D) Top 15 upregulated (C) and downregulated (D) KEGG pathways associated with differentially expressed genes under the same treatment conditions, ranked by −log_10_(adjusted *P*-value).

To further explore the molecular mechanisms underlying the antiviral effects of **DK@RA-PEG**, we performed RNA-seq analysis on RABV-infected BSR cells after treatment with **DK@RA-PEG**. The treatment with **DK@RA-PEG** triggered significant transcriptomic changes compared to the noninfected group. Specifically, among 1663 differentially expressed genes (DEGs) (adjusted *P*-value < 0.05, |log_2_ fold change| > 0.5), 811 genes were upregulated, while 852 genes were downregulated (Figure 4A). Pathway enrichment analysis based on the Kyoto Encyclopedia of Genes and Genomes (KEGG) database revealed that these DEGs were predominantly involved in fluid shear stress and atherosclerosis, metabolism-related signaling pathways, and ROS-related signaling pathways, such as focal adhesion, the JAK-STAT signaling pathway, lysosome function, apoptosis, and glutathione metabolism (Figure 4B). Notably, KEGG analysis of the upregulated genes showed significant enrichment in the peroxisome-related processes and metabolic-related pathways (Figure 4C). In contrast, analysis of the downregulated genes highlighted significant enrichment in pathways, such as lysosome, JAK-STAT signaling, apoptosis, NF-κB signaling, and glutathione metabolism (Figure 4D). The observed reduction in glutathione metabolism and decreased focal adhesion, lysosome, and apoptosis collectively suggest that the treatment of RABV-infected BSR cells with **DK@RA-PEG** activates ROS-related processes. Furthermore, downregulation of the NF-κB signaling pathways indicates a dampened inflammatory response. Altogether, these results suggest that PDT treatment with **DK@RA-PEG** not only stimulates oxidative stress, potentially aiding in the clearance of RABV, but also mitigates the intracellular inflammatory response triggered by RABV infection.

### **DK@RA-PEG** Exhibits *In Vivo* BBB Crossing and Brain-Targeting Capability

**Figure 5:**
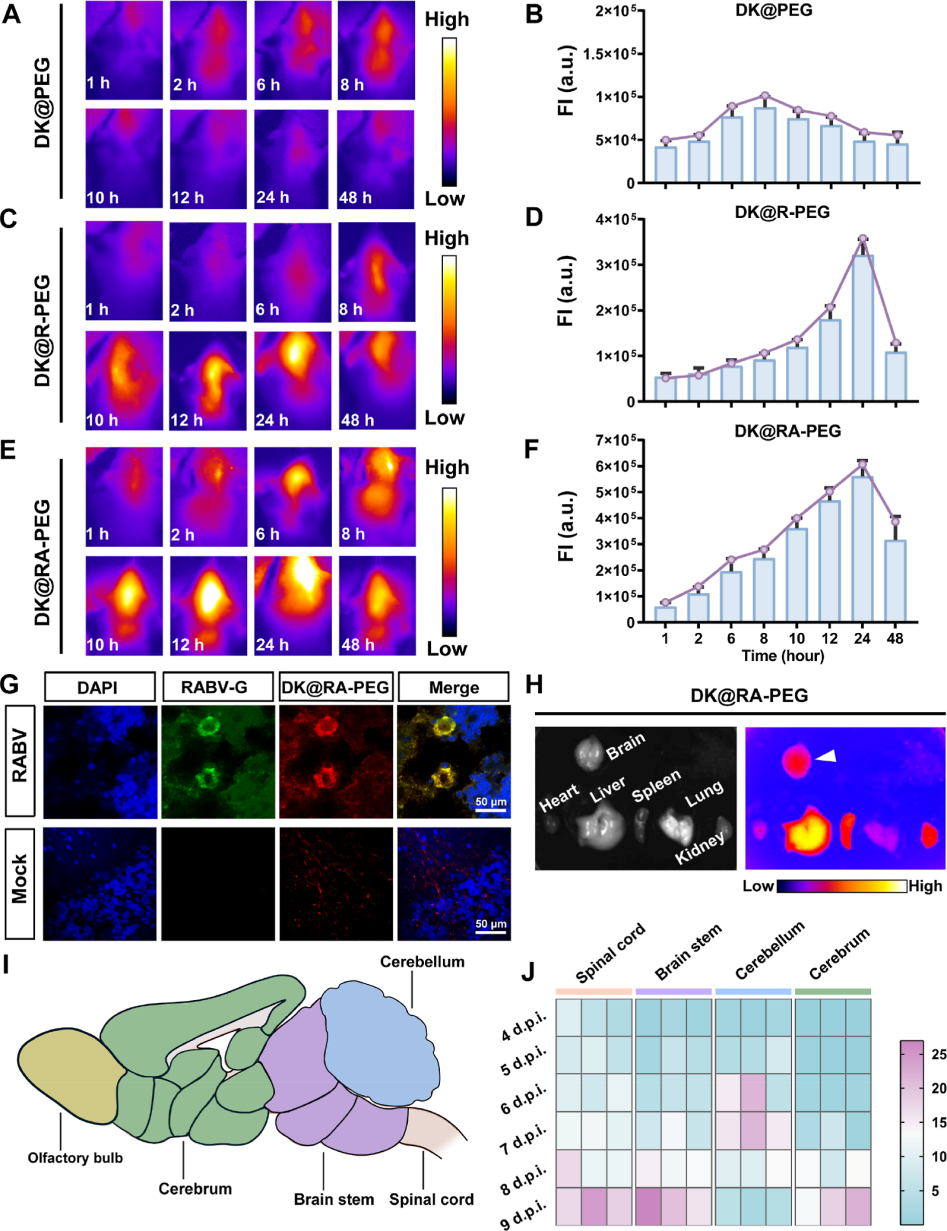
Targeting ability and drug light interval of **DK@RA-PEG***in vivo*. (A,C,E) The representative NIR-II images (808 nm excitation, 90 W/cm^2^, 1000 nm LP, 300 ms) of different time points after i.v. administration of DK@PEG (A), DK@R-PEG (C), and **DK@RA-PEG** (E) *in vivo*, respectively. (B,D,F) The FI of DK@PEG (B), DK@R-PEG (D), and **DK@RA-PEG** (F) were analyzed by ImageJ (*n* = 3). The curves represent the highest values from three replicate experiments. FI, fluorescence intensity. (G) Co-localization of RABV-G (green) and **DK@RA-PEG** (red) in mouse brains from RABV-infected mice at 7 dpi was confirmed by the immunofluorescence assay. The sections were stained with RVG-specific mAb, Alexa Fluor 488 goat anti-mouse IgG, and DAPI. Scale bar, 50 μm. (H) Tissue distribution of **DK@RA-PEG** in the mice. The fluorescence signal was detected by the NIR-II IVIS spectrum imaging system in different tissues (heart, liver, spleen, lung, kidney, and brain) from Balb/c mice inoculated with **DK@RA-PEG** via the i.v. route at 24 hpi. (I) A parasagittal view of the whole mouse brain. Major divisions were labeled with chartreuse (olfactory bulb), green (cerebrum), purple (brain stem), blue (cerebellum), and pink (spinal cord). (J) RABV infection kinetics in the central nervous system of mice after i.m. challenge. The heat maps were established based on the fold change in mRNA expression of RABV-N in different brain areas (spinal cord, brain stem, cerebellum, and cerebrum) at indicated time points (4, 5, 6, 7, 8, and 9 dpi). For each sample, the mRNA abundance (CT value) of the target genes was normalized to that of the endogenous β-actin reference gene. The fold change in gene expression was further calculated using 2^–ΔΔ*CT*^. Data in (B), (D), and (F) are represented as the mean ± SD.

The inability of potential drug molecules to cross the BBB is a major reason why rabies is difficult to cure. Therefore, increasing the brain targeting and permeability of drugs is an important requisite for the treatment of rabies. To evaluate whether **DK@RA-PEG** could cross the BBB *in vivo*, 6 days after RABV infection, we used the IVIS spectrum *in vivo* imaging system to monitor brain aggregation within 48 h after intravenous (i.v.) injection of **DK@RA-PEG**. In addition, **DK@PEG** (unmodified RVG peptide and aptamer) and **DK@R-PEG** (modified only RVG peptide) were used as controls. In this experiment, we observed that both **DK@RA-PEG** and **DK@R-PEG** could effectively cross the BBB and accumulate in the brain, reaching a peak at 24 hpi (Figure 5C–F). However, within 12–48 hpi, the fluorescence signals from the **DK@RA-PEG** group were twice as high as for **DK@R-PEG** (Figure 5D,F). In contrast, **DK@PEG** without RVG modification did not cross the BBB (Figure 5A,B). These results demonstrate that **DK@RA-PEG** can be successfully delivered to the CNS and reaches a peak of BBB permeability around 24 h. Additionally, strong fluorescence signals could be observed in the brain, liver, spleen, and kidneys in the **DK@RA-PEG**-treated group, while no obvious fluorescence signals were observed in the heart and lung (Figure 5H). To further verify whether **DK@RA-PEG** could target RABV-infected neurons in the CNS, we intravenously injected **DK@RA-PEG** 6 days post-RABV infection and collected brain tissues 24 h later for immunofluorescence staining. As shown in Figure 5G, we found that RVG and **DK@RA-PEG** were colocalized in brain tissues, suggesting that **DK@RA-PEG** can target RVG in infected neurons. These results indicate that **DK@RA-PEG** exhibits good BBB-crossing *in vivo* and is a suitable platform for the delivery of therapeutic entities for the treatment of rabies.

### **DK@RA-PEG** Exhibits Robust *In Vivo* Activity against RABV

To investigate the therapeutic activity of **DK@RA-PEG** against lethal RABV infection *in vivo*, we established a Balb/c mouse muscle infection model using a fixed RABV strain, CVS-B2c. In this model, we first detected viral kinetics in brain tissue after RABV infection by a qRT-PCR assay. As shown in Figure 5I,J, total RNA was extracted from the harvested spinal cord, cerebellum, brainstem, and cerebrum tissues (including olfactory bulb) from 4 to 9 days after infection. The viral load in each region was quantified by measuring the mRNA levels of the RABV nucleoprotein (RABV-N). Our results indicated that RABV was detected from 4 dpi in the spinal cord, 5 dpi in the brainstem and cerebellum, and 6 dpi in the cerebrum (Figure 5J and Table S2).

**Figure 6:**
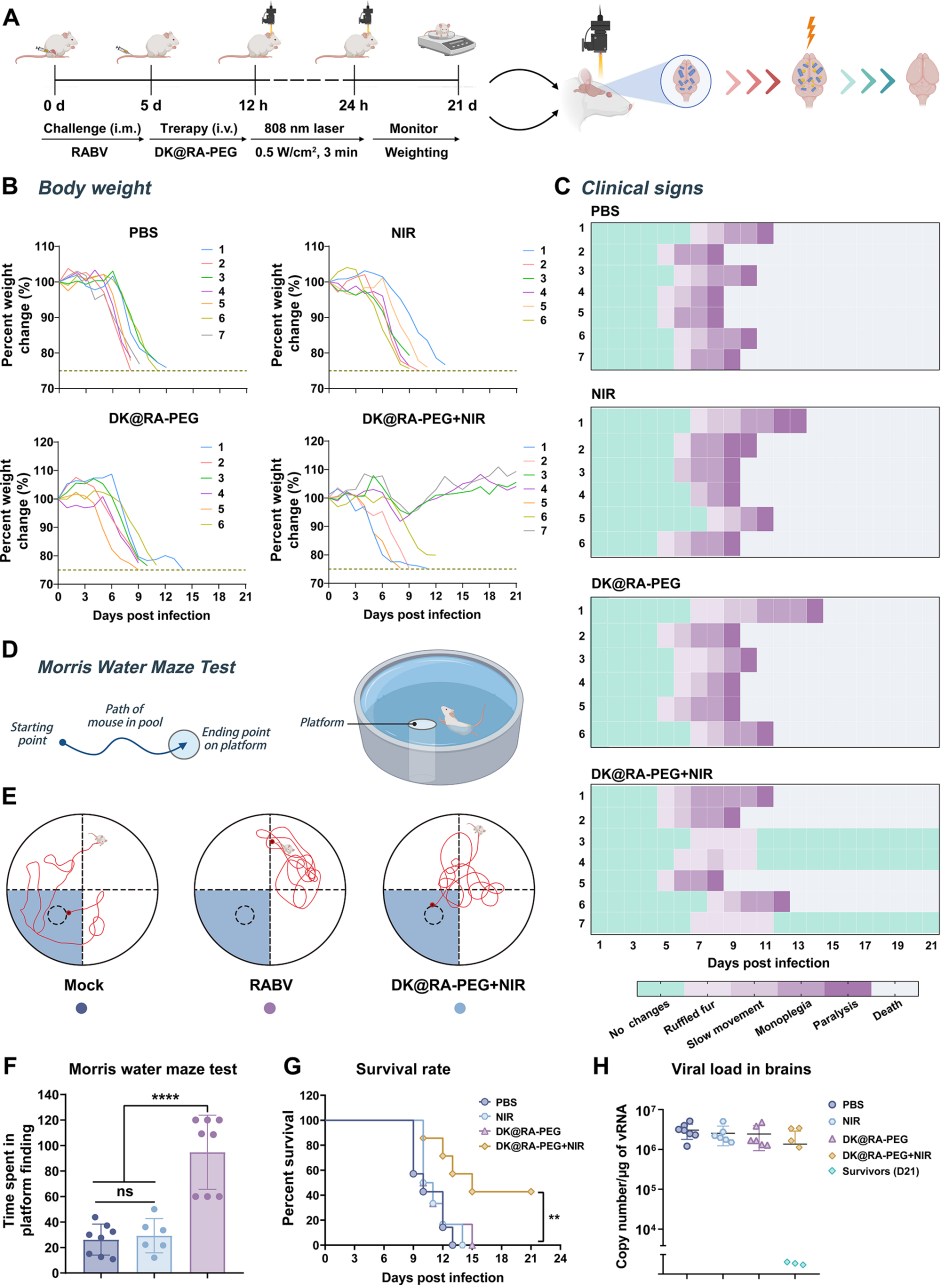
Antiviral activity of **DK@RA-PEG***in vivo*. (A) Schematic illustration of DK@RA-PEG-mediated PDT in the mouse model. Balb/c mice were infected with RABV by i.m. injection at 0 dpi and treated with **DK@RA-PEG** by i.v. injection at 5dpi, respectively. Then, mice were irradiated by NIR laser (808 nm, 0.5 W/cm^2^) for 3 min at 12 and 24 h after injection of DK@RA-PEG. (B) The body weight changes of mice were recorded daily for 21 days under four different treatments (*n* = 6 or 7). (C) The clinical signs in mice for 21 consecutive days under four different treatments. Heat maps were established based on a progressive 0–10 clinical score scale (0–1: no changes; 2–3: ruffled fur; 4–5: slow movement; 6–8: monoplegia; 9: paralysis, tremors; 10: death). Each row represents one animal throughout time. (D) The general procedures for the Morris water maze (MWM) test. (E) Schematic diagram for the MWM test and the representative swimming track on evaluation day. (F) Time spent in platform finding of each group on the probe test day. (G) Cumulative Kaplan–Meier survival curves of mice under four different treatments were recorded for 21 days. (H) Genomic RNA copy numbers of RABV in mouse brains from four groups were quantified by qPCR at 8 or 21 dpi (*n* = 6 or 7). Data are represented as the mean ± SD. Statistical significance was calculated by one-way ANOVA with Tukey’s multiple comparisons test in (F) and the log-rank (Mantel–Cox) test in (G). **P < 0.01, ****P < 0.0001, ns, not significant.

Based on the viral dynamics and the drug-light interval of **DK@RA-PEG**, we performed *in vivo* experiments as illustrated in Figure 6A. Briefly, **DK@RA-PEG** was i.v. injected 5 days after the RABV challenge and then irradiated with an 808 nm NIR laser (0.5 W/cm^2^) 12 and 24 h later. The body weight and clinical symptoms of mice were monitored for 21 consecutive days from the start of the challenge. In these experiments, we found that 3 out of 7 mice survived after the PDT treatment with **DK@RA-PEG**, whereas all mice in the other three groups died within 14 dpi. These remarkable results indicate increased survival rates by around 40% and confirm that neither **DK@RA-PEG** nor light irradiation on their own could effectively eliminate RABV *in vivo* (Figure 6G). Furthermore, only 5% weight loss was observed in PDT-treated mice during 6–9 days of dpi, and the weight returned to normal levels after 13 dpi. In contrast, mice in the non-PDT group lost more than 25% of their body weight from 5 dpi, showed decreased motor ability from 7 dpi, and began to show typical clinical symptoms (ruffled fur, lethargy, ataxia, and paralysis) from 8 dpi (Figure 6B,C).

In order to verify whether RABV in the brain of the PDT-treated mice was completely cleared, we harvested brain tissue from mice after PDT treatment at 21 dpi for viral genomic RNA quantification. For the other groups, brain tissue was collected at the time of death or when their body weight dropped to 75%. The results of qPCR showed that no virus was detected in the brain tissues of the surviving mice (Figure 6H). The viral load in various brain regions of mice was further determined by immunohistochemical analysis. No RABV-P-positive cells were observed in the cerebral cortex, brainstem, and cerebellum of surviving mice (Figure S30A–E). In contrast, the numbers of RABV-P-positive cells in the other three groups were significantly higher (Figure S30A–E). Consistent with this, hematoxylin and eosin staining showed that vascular cuffs and hyperplasia were visible in the brains of mice in the RABV, **DK@RA-PEG,** or NIR groups, while no obvious pathological lesions were observed in the brains of mice in the PDT-treated group (Figure S31).

Finally, we performed the Morris water maze test to evaluate the spatial learning, memory ability, and sense of direction of PDT-treated mice.^23^ For mice that survived after PDT with **DK@RA-PEG**, we performed a platform-finding test at 21 dpi and after a 5-day training. Mice in the RABV control group began training 1 day after infection and were tested at 6 dpi. The results showed that the time spent by the surviving mice finding the platform was not significantly different from that of the blank control group, indicating that PDT treatment did not have a significant effect on the behavior of mice (Figure 6D–F). On the contrary, RABV-infected mice needed thrice as long to find the platform compared to the blank control group, and even 3 mice failed to find the platform within the specified time (Figure 6D–F). This data indicate that the learning and memory abilities of mice had been severely affected 6 days after infection. In addition, no obvious tissue inflammation was observed in the brains of cured mice (Figure S32). Simultaneously, no obvious abnormalities were found in histopathology, hepatic and renal function, and blood biochemical analysis after **DK@RA-PEG** injection (Figures S33 and S34), indicating that **DK@RA-PEG** has superior safety and biocompatibility *in vivo*.

## Conclusions

In this study, we successfully developed **DK@RA-PEG** as a novel NIR-II nanotheranostic probe that effectively penetrates the BBB, targets RVG, and facilitates both high-resolution imaging and PDT for RABV infections *in vivo*. Our findings demonstrate that **DK@RA-PEG** can precisely localize and eliminate RABV-infected cells in the CNS with minimal off-target effects. The use of NIR-II imaging significantly enhances tissue penetration, while the exclusive generation of ROS without thermal effects ensures safe PDT. **DK@RA-PEG** showed excellent biocompatibility, safety, and efficacy both *in vitro* and *in vivo* with remarkable improvements in survival rates and neurological function in RABV-infected mice following PDT treatment. These results, further supported by transcriptomic analysis, highlight the potential of our chemical platform to accelerate therapeutic development against rabies infections, particularly in the absence of postsymptomatic treatments. Our work opens new avenues for the application of NIR-II-guided PDT in CNS-targeted diseases, offering a promising approach for overcoming the challenges of BBB crossing and the complexity of neurotropic viral infections. Future studies will focus on optimizing this platform for clinical translation to advance the currently limited therapeutic options for rabies and other CNS infections.

## Supporting Information Available

Materials, apparatus, and additional analytical data (PDF)
