# Supplementary material for: Rabies Virus Targeting NIR-II Phototheranostics
Source: J Am Chem Soc. 2025 May 2;147(19):16661–73. doi: 10.1021/jacs.5c04975 (PMC12082629; doi:10.1021/jacs.5c04975)
Supplement: Supplementary file 1 — ja5c04975_si_001.pdf [file ja5c04975_si_001.pdf]

Rabies Virus Targeting NIR-II Photo-Theranostics

Qihang Ding,[a]+ Caiqian Wang,[b]+ Haoran Wang,[b] Chunbai Xiang,[c] Zhao Wang,[d] Yue Wang,*[c] Ling Zhao,*[b] Marc Vendrell,*[e] Jong Seung Kim*[a]

[a]Department of Chemistry, Korea University, Seoul 02841, Korea

[b]National Key Laboratory of Agricultural Microbiology, Huazhong Agricultural University, Wuhan 430070, China

[c]Guangdong Key Laboratory of Nanomedicine, CAS-HK Joint Lab for Biomaterials Shenzhen Institutes of Advanced Technology, Chinese Academy of Sciences, Shenzhen 518055, China

[d]Oujiang Laboratory (Zhejiang Lab for Regenerative Medicine, Vision, and Brain Health), Institute of Aging, Key Laboratory of Alzheimer's Disease of Zhejiang Province, The Second Affiliated Hospital, Wenzhou Medical University, Wenzhou 325000, China

[e]Centre for Inflammation Research and IRR Chemistry Hub, Institute for Regeneration and Repair, The University of Edinburgh, Edinburgh EH16 4UU, United Kingdom

Materials and methods

Materials. All chemicals used in this study were obtained from reputable commercial suppliers, including Aldrich, Energy Chemical, and TCI, and were employed without additional purification unless explicitly stated otherwise. UV-Vis-NIR absorption spectra were recorded on a Shimadzu UV-3600 spectrophotometer. NIR-II fluorescence measurements were carried out using a Fluorolog 3 spectrofluorometer (Horiba) equipped with an 808 nm diode laser. MALDI-TOF-MS analysis was conducted using a Bruker Autoflex Speed MALDI-TOF mass spectrometer. NIR-II fluorescence imaging was performed on a dedicated imaging system (Wuhan Grand Imaging Technology Co., Ltd). Transmission electron microscopy images were acquired using an HT7700 transmission electron microscope operating at an acceleration voltage of 100 kV. Confocal fluorescence imaging was conducted using a confocal laser scanning microscope in conjunction with a two-photon in vivo imaging system and a Leica SPE microscope.

Cells, viruses and antibodies, and animals. BSR cells (a clone of BHK-21) and N2a (mouse neuroblastoma) were cultured in Dulbecco modified Eagle's medium (DMEM, BioChannel Biotechnology Co., Ltd) containing 10% fetal bovine serum (FBS, QmSuero Biotech Co., Ltd.) and 1% penicillin/streptomycin (Biosharp). CVS-B2c was obtained after continuous passage of the CVS-24 strain[1]. DRV-Mexico is a wild-type RABV isolated from a rabid dog[2]. SHBRV is a bat-derived RABV strain isolated from a human patient[3]. These three strains are stored in our laboratory. A monoclonal antibody (mAb) against RABV nucleoprotein (RABV-N) and a monoclonal antibody (mAb) against RABV glycoproteins (RVG) was prepared in our laboratory. The mAb against phosphoprotein (RABV-P) was conjugated with FITC (FITC-RABV-P) by Friendbio Science & Technology Co.Ltd. (Wuhan, China). A mAb against β-actin was purchased from ProteinTech (Proteintech Group, Inc., USA). Balb/c mice were purchased from the Hubei Center for Disease Control, Wuhan, China, and raised in the Animal Facility at Huazhong Agricultural University. All experimental procedures were approved by the Scientific Ethics Committee of Huazhong Agricultural University (permit number: HZAUMO-2023-0181).

Cytotoxicity assays. The cytotoxicity of DK@RA-PEG was analyzed using the containing cell counting Kit-8 (CCK-8, Abbkine) method following the manufacturer's instructions. Briefly, BSR or N2a cells were seeded into 96-well plates with 1×10^4 cells per well. Then, the monolayers of BSR or N2a cells were incubated with 100 µL DMEM (2% FBS) containing different concentrations (0, 31.25, 62.5, 125, 250, 500, 1000, 2000 nM) of DK@RA-PEG at 37 °C for 36 h. Next, the supernatant was removed and replaced by fresh DMEM containing CCK-8. After incubation for 2 h, the viability of cells was measured by detecting absorbance at 450 nm using a SpectraMax190 spectrophotometer (Molecular Devices, CA, USA).

Viral inhibition assays. To evaluate the antiviral efficacy of DK@RA-PEG *in vitro*, BSR and N2a cells were cultured overnight in a 24-well cell-culture petri dish with a density of 2 x 10^5 cells/well. Then, the cells were infected with RABV (CVS-B2c, DRV-Mexico, or SHBRV) at an MOI of 0.01 for 1 h at 37 °C. Next, the supernatant was removed, and cells were further cultured with fresh medium containing different concentrations of DK@RA-PEG and irradiation with the NIR (808 nm, 0.5 W/cm^2) for 1 min. The no laser irradiation group was set up simultaneously. At 48 hpi, the cell supernatant was collected for virus titration, and cells were lysed with RIPA buffer (Beyotime Biotech Inc., China) and TRIzol® Reagent (Invitrogen Inc., California, USA) for further analysis.

Immunofluorescence analysis. In order to observe the expression of intracellular viral proteins after **DK@RA-PEG** treatment, the supernatant was removed, and the cells were washed 3 times with PBS after 48 hours of drug incubation. Then, the cells were fixed with 4% paraformaldehyde for 15 min and permeabilized in PBS (0.1% Triton X-100). After the cells were washed 3 times with PBS, the FITC-RABV-P was incubated with cells at 37 °C for 30 min. Cell images were taken with a DP80/BX53 fluorescence microscope. To investigate the targeting ability of DK@RA-PEG *in vitro*. N2a cells were transfected with plasmids encoding glycoproteins of different RABV strains (RABV-CVS, RABV-DRV, and RABV-SHBRV), and blank pCAGGS plasmid was used as a negative control. After 24 h, cells were incubated with DK@RA-PEG for 12 hours and then incubated with RVG-specific mAb overnight at 4 °C. After washing three times, the cells were further incubated with Alexa Fluor™ 488 Goat anti-Mouse IgG (H+L) secondary antibody (Invitrogen) for 1 h. Nuclei were stained

with 2-(4-Amidinophenyl)-1H-indole-6-carboxamidine (DAPI, Beyotime) for 10 min. Then imaging was performed on a Nikon A1HD25 super-resolution laser scanning confocal microscopy. To evaluate the targeting effect of DK@RA-PEG *in vivo*, Balb/c mice were intravenously injected with DK@RA-PEG at 6 days after infection with RABV. Brain tissues were collected 48 hours later and embedded into an O.C.T. Compound (SAKURA). Then, the brain tissues were flash-frozen and sectioned into 20 μm slices using a CM1950 cryostat (Leica, Heerbrugg, Switzerland). Sections were blocked in PBS supplemented with 10% concentrated goat serum (Boster) for 2 h at room temperature. Next, sections were incubated with anti-RABV-G mAb overnight at 4 °C. After washing three times, the sections were further incubated with Alexa Fluor™ 488 Goat anti-Mouse IgG (H+L) secondary antibody for 1 h. Images were captured with a Nikon AXR NSPARC super-resolution laser scanning confocal microscopy.

Virus titration. The cell supernatant was titrated by using the direct fluorescent antibody (DFA) assay as described previously[4]. Briefly, BSR cells in 96-well plates were inoculated with serial 10-fold dilutions of supernatant containing RABV and incubated for 48 h at 37 °C. After incubation, cells were fixed with 80% ice-cold acetone and then stained with FITC-RABV-P antibody. The fluorescence antigen-positive foci were counted using an IX51 fluorescence microscope (Olympus, JPN), and viral titers were calculated as focus-forming units/mL (FFU/mL).

Quantitative reverse transcription-PCR (qRT-PCR). Total RNA was isolated from cells or brain tissues using TRIzol® Reagent (Thermo, USA) according to the manufacturer's instructions. cDNA was generated with equal amounts of total RNA from each sample by using HiScript® II Q RT SuperMix and HiScript® II 1st Strand cDNA Synthesis Kit (Vazyme Biotech Co., Ltd, China). Then qRT-PCR was performed using Universal SYBR® Green Supermix (Bio-Rad, USA). The primers for RABV-specific viral RNA, RABV-N mRNA, and mouse β-actin are shown in Table S1. The transcript levels of genomic RNA and targeted genes were calculated relative to the levels of β-actin using the formula: $2^{-\Delta\Delta CT}$. A standard curve was generated from serially diluted plasmids (pcDNA3.1-CVS-B2c), and the copy number of RABV-vRNA was normalized to 1 μg of total RNA.

Western blot analysis. Cells were washed with PBS and lysed in RIPA buffer (Beyotime) with 1mM phenylmethylsulphonyl fluoride (PMSF). Protein concentrations were determined with a BCA Protein Assay kit (Beyotime). An equal amount of protein lysate was separated by 10% SDS-polyacrylamide gels and transferred to PVDF membranes (Bio-Rad). Membranes were blocked with TBST with 5% (w/v) non-fat dry milk for 4 h at room temperature and probed with the antibody against RABV-N or β-actin overnight at 4 °C. After washing 3 times with TBST, PVDF membranes were probed with HRP-conjugated goat anti-mouse IgG antibody (Boster, China) for 1 h at room temperature. Signals were acquired and analyzed using a Clarity Western ECL Substrate (Bio-Rad, USA).

***In vivo* NIR-II imaging.** Six-week-old female Balb/c mice were randomly divided into 3 groups (DK-PEG, DK@R-PEG, DK@RA-PEG) and were i.m. injected with 6×10^5 FFU RABV-CVS-B2c. At 7 dpi, mice were intravenously injected with DK-PEG, DK@R-PEG, or DK@RA-PEG. The real-time fluorescent signals in the brain areas were recorded by the Lumina IVIS III imaging system (Per-kin/Elmer) at the indicated time point. The excitation wavelengths are 808 nm. The fluorescence signal intensity was analyzed using imageJ software.

Antiviral effect of DK@RA-PEG *in vivo*. All mice were acclimatized for one week prior to formal experiments, and mice that were overweight or underweight were excluded to reduce experimental errors caused by environmental factors and human intervention. Balb/c mice of appropriate weight were randomly divided into 4 groups (PBS, DK@RA-PEG, NIR, DK@RA-PEG+NIR) and were i.m. injected with 4×10^5 FFU RABV-CVS-B2c. At 5 dpi, the DK@RA-PEG and DK@RA-PEG+NIR groups were i.v. injected with 0.2 mg mL^{-1} DK@RA-PEG. The DK@RA-PEG+NIR group was irradiated with laser (808 nm, 0.5 W/cm^2) for 3 min at 12 h and 48 h after administration. The NIR control group was also irradiated at the same time points. The weight change, clinical symptoms, and mortality were observed once a day for 21 days. The evaluation of clinical symptoms adopts the scoring system, that is, 0-1: no changes; 2-3: ruffled fur; 4-5: slow movement; 6-8: monoplegia; 9: paralysis, tremors; 10: death.

Histology. Major tissues of Mice in different groups (PBS, DK@R-PEG, and DK@RA-PEG), which

included heart, liver, spleen, lung, and kidney were collected and fixed in 4% paraformaldehyde buffer (Biosharp). The brain samples were either collected at the time of death, or at 21 dpi for the survivors and fixed in 4% paraformaldehyde buffer for 48 h. After fixation, brain tissue was embedded in paraffin and cut into 4-5 μm thick sections along the sagittal plane. The sections were stained with hematoxylin and eosin (H&E) to detect cell lesions. For immunohistochemistry (IHC), brain sections were blocked for endogenous peroxidase in 30% hydrogen peroxide for 20 min and antigens were retrieved in citrate buffer[5]. Then, sections were incubated with rabbit anti-RABV-P antibody and rabbit anti-CD45 antibody to detect the expression level of viral antigen and inflammation.

Morris water maze (MWM) tests. Mice were subjected to an MWM test to monitor spatial cognition, learning, and memory 1 day after RABV infection or at 21 dpi for the survivors as previously described[6]. Briefly, all mice were trained and tested in a black, 1.5-m-diameter water maze containing four quadrants. The maze was filled with water to a depth of approximately 0.25 m and dyed black with ink, and a 10-cm-diameter escape platform was fixed 2 cm above the water surface in the target quadrant. A camera was set up above the pool and connected to a computer tracking system. During the first five days of the learning phase, mice received three training sessions per day for 120 s each. The training content was that the mouse was placed in a random position facing the wall of the other three quadrants, and the movement trajectory of the mouse was recorded. If mice could find the platform in the water within 120 s, they were allowed to stay on the platform for 5 s to adapt to the surrounding environment; if the mice could not find the platform within 120 s, they were guided to the platform and stayed for 5 s. After the training, the mice were taken out of the water, wiped with a towel, and placed in an incubator. Then the mice were placed in the cage when the mice were thoroughly dried. During the test phase (day 6), mice were placed in the opposite position of the target quadrant, the platform was removed, and the mice were allowed to swim freely for 120 s. The movement trajectory of the mouse and the time required to reach the platform were recorded by a computer. The time for mice that failed to find the platform was recorded as 120 s.

Transcriptome sequencing and bioinformatic analysis. BSR cells infected with RABV and treated with DK@RA-PEG+NIR were collected, and total RNA was extracted using the TRIzol® Reagent

(Thermo, USA). RNA sequencing (RNA-Seq) analysis was conducted at OE Biotech, Inc., (Shanghai, China). Briefly, RNA integrity was assessed using an Agilent 2100 Bioanalyzer (Agilent Technologies, Santa Clara, CA, USA) before the construction of cDNA libraries. Samples with an RNA integrity number (RIN) ⩾ 7 were subjected to subsequent analysis. Then, cDNA libraries were constructed and sequenced on the Illumina Novaseq 6000 platform and 150 bp paired-end reads were generated. The raw sequencing data in FASTQ format were preprocessed using fastp (v0.23.4)[7], where adapter sequences and low-quality reads were removed to yield high-quality clean reads. These clean reads were then mapped to the reference genome (*Mus musculus*) using HISAT2 (v2.2.1)[8] with default parameters. Fragments Per Kilobase of transcript per Million mapped reads (FPKM) of each gene were calculated, and gene read counts were obtained using HTSeq (v0.11.1)[9, 10]. Differential gene expression analysis was performed with these read counts using the Bioconductor package DESeq2 (v1.28)[11]. Fold Change is also calculated in DESeq2 (v1.28). Genes with an adjusted *P*-value < 0.05 and $|Log_2Fold\ Change| > 0.5$ were selected as differential expression genes (DEGs). Functional annotation and pathway enrichment analyses, including KEGG pathway and GSEA analyses, were performed using the Bioconductor package clusterProfiler (v3.8)[12].

Statistical analysis. All data were analyzed using GraphPad Prism 8.0 software (GraphPad Software Inc., CA, USA) and presented as the mean standard deviations (SD). Statistical significance was determined by using unpaired two-tailed t-test, one-way or two-way ANOVA. The significance of the differences in all analyses was expressed as: *, P<0.05; **, P<0.01; ***, P<0.001 or **** P<0.0001.

Data availability

All relevant data are available in the main text and Supporting Information and can be obtained from the authors upon request.

Supplementary Figures

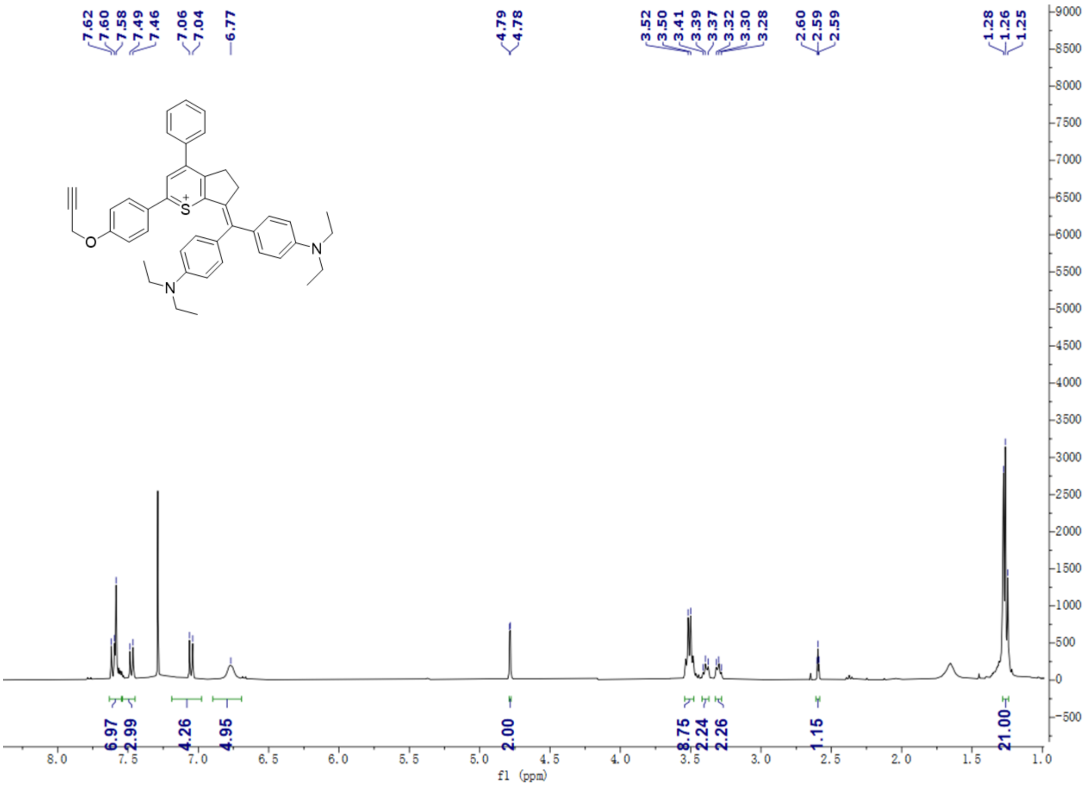

Figure S1. ^{1}H-NMR spectrum of DK in $CDCl_3$.

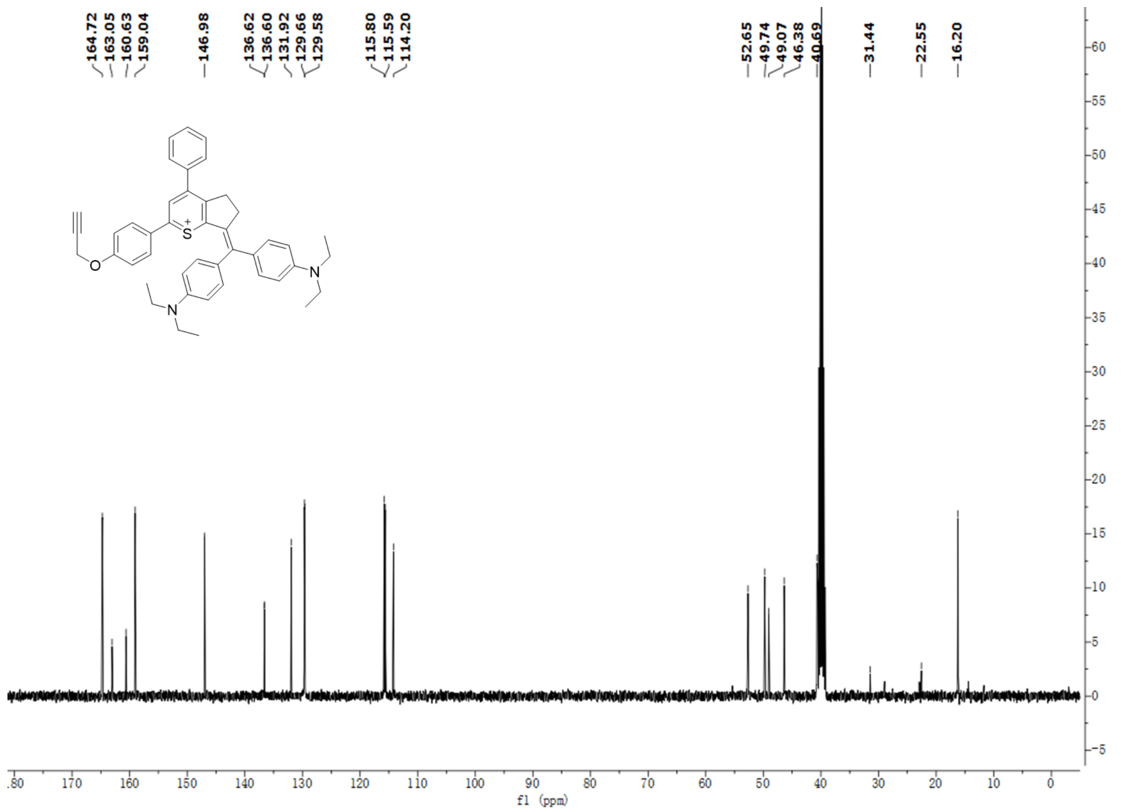

Figure S2. ^{13}C-NMR spectrum of DK in $CDCl_3$.

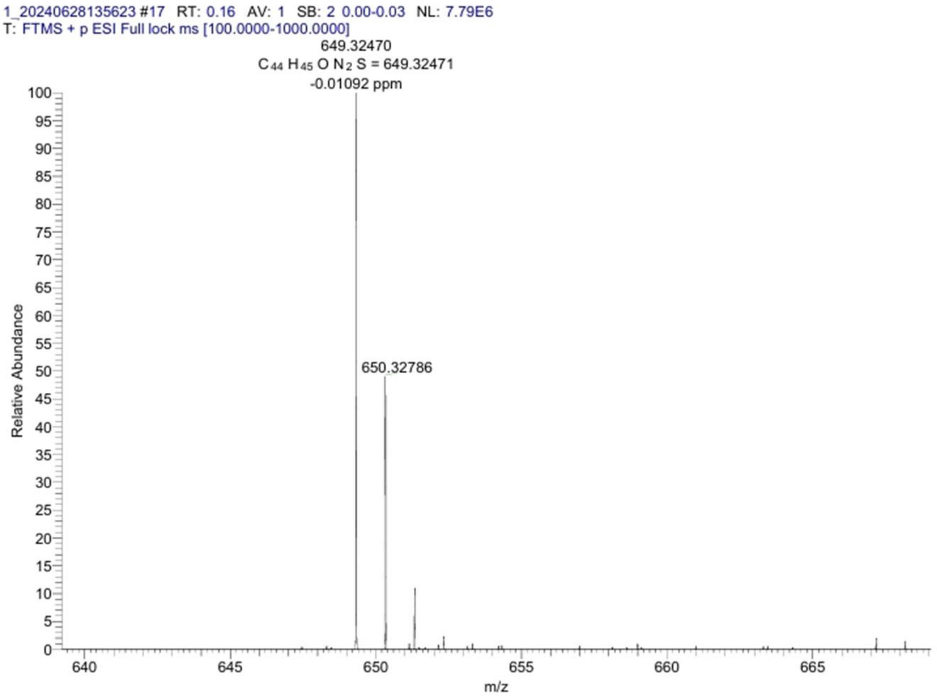

Figure S3. HRMS spectrum of DK.

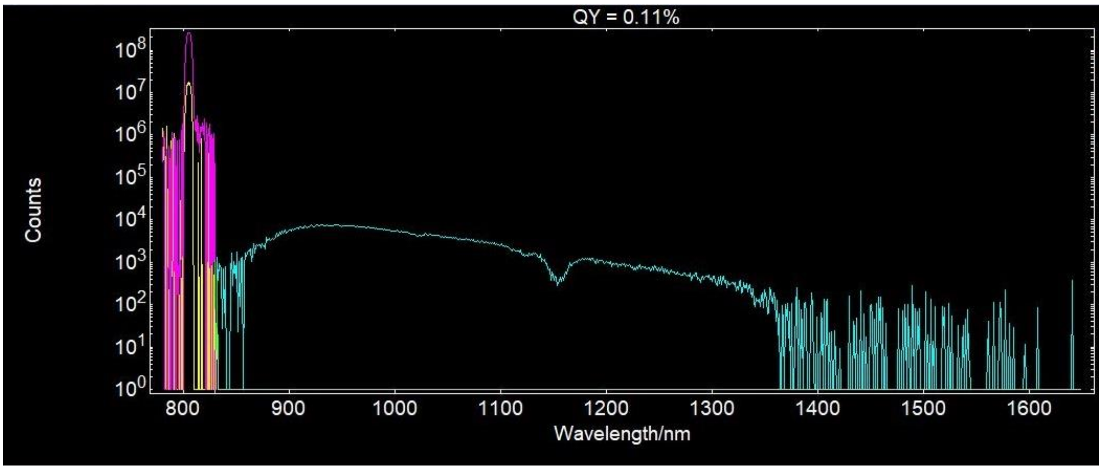

Figure S4. Fluorescence quantum yield (Φ) of DK measured under the conditions of 808 nm laser irradiation (0.5 W/cm^2) (Φ: 0.11%. Concentration: 0.2 mg/ml in DCM).

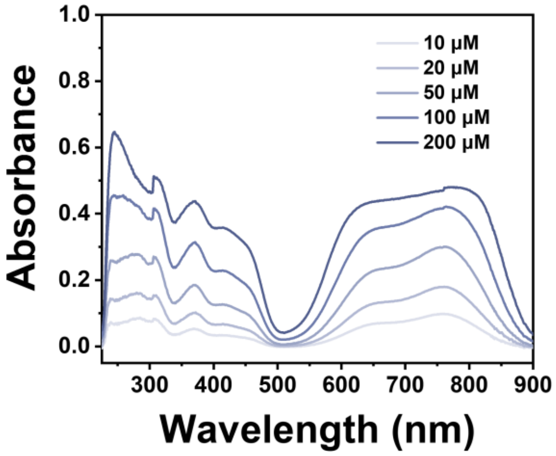

Figure S5. Absorbance of DK in DCM with different concentrations.

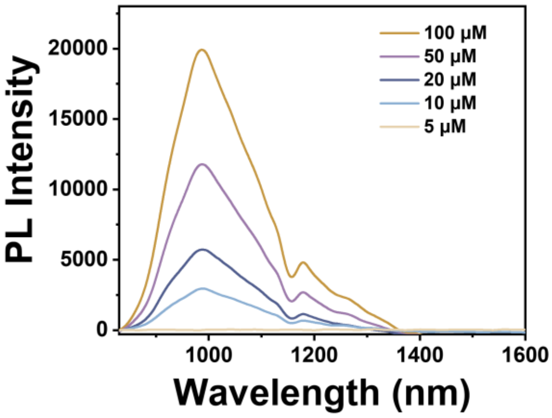

Figure S6. Fluorescence emission of DK in DCM with different concentrations.

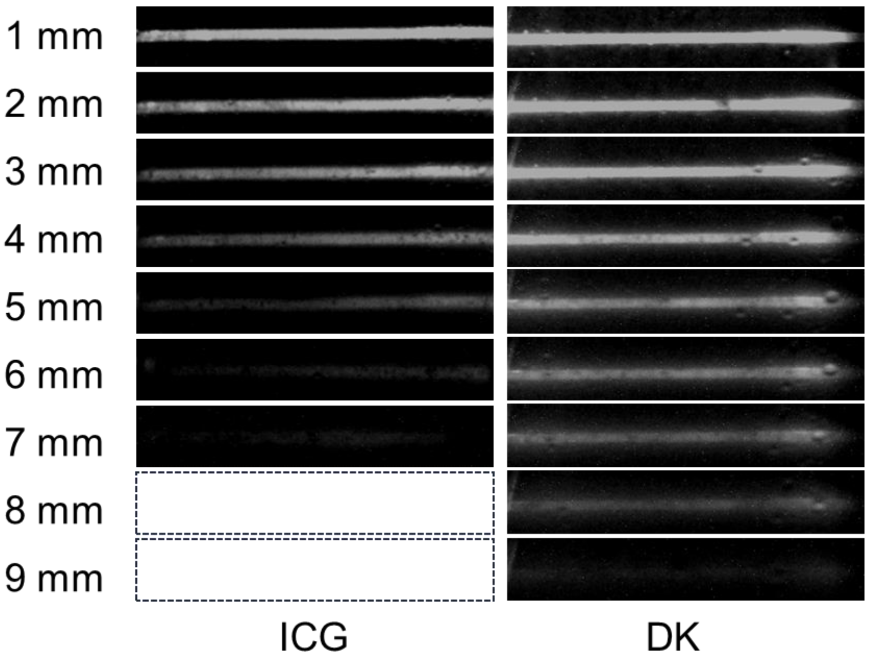

Figure S7. *In vitro* penetration depth in the NIR-II window (1000 nm Long-pass filter) using intralipid as tissue phantoms imitator.

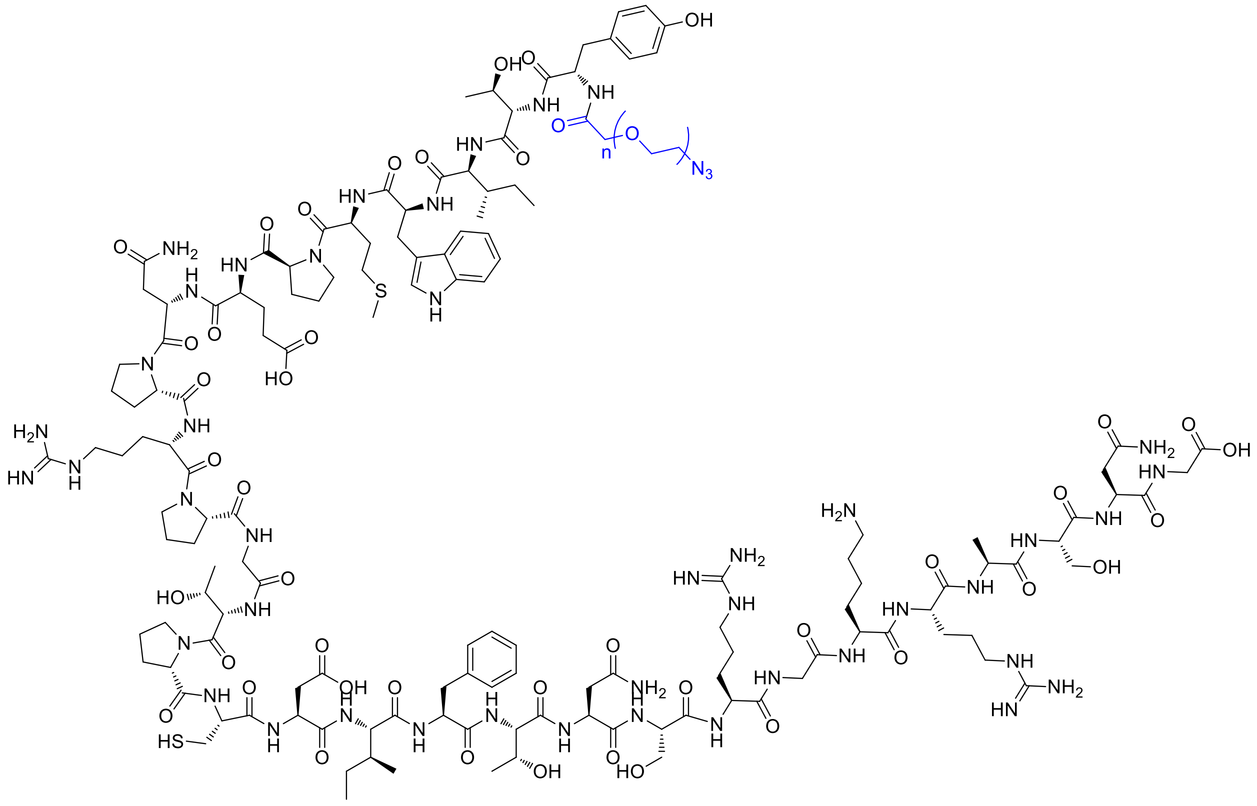

Figure S8. Chemical structure of N3-PEG2000-YTIWMPENPRPGTPCDIFTNSRGKRASNG.

Sequence: N3-PEG2000-YTIWMPENPRPGTPCDIFTNSRGKRASNG

Column: 4.6*150mm, kromasil C18-5

Solvent A: 0.1%Trifluoroacetic in 100% Acetonirile

Solvent B: 0.1%Trifluoroacetic in 100% Water

Gradient:		A	B
	0.01min	5%	95%
	25.0min	75%	25%
	30min	90%	10%

Flow rate: 1.0ml/min Wavelength: 214nm Volume: 20ul

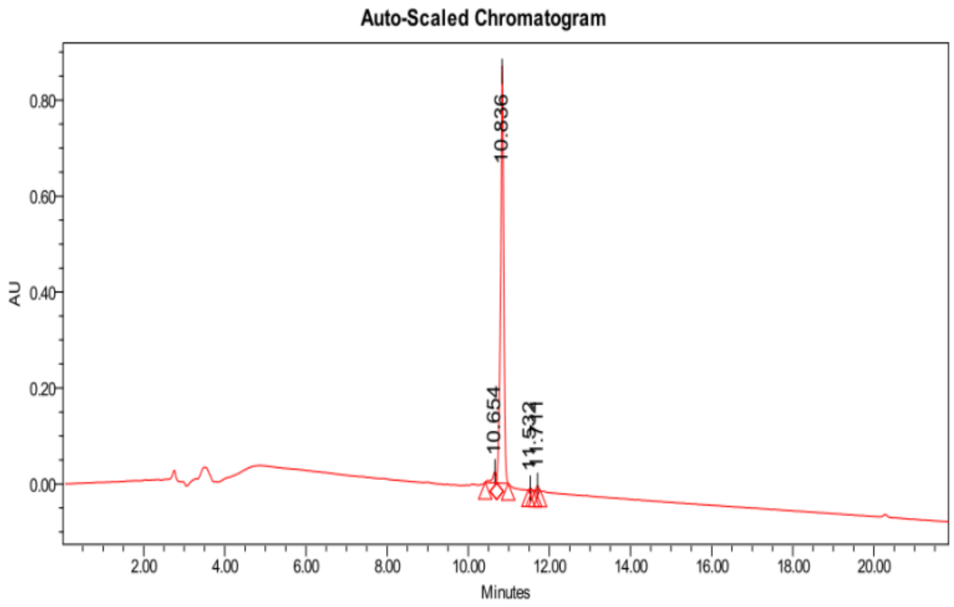

Peak Results

	RT	Area	Height	% Area
1	10.654	147094	22495	3.09
2	10.836	4561270	852251	95.85
3	11.532	12175	3533	0.26
4	11.71[illegible]	38002	9801	0.80

Figure S9. HPLC analysis of N3-PEG2000-YTIWMPENPRPGTPCDIFTNSRGKRASNG.

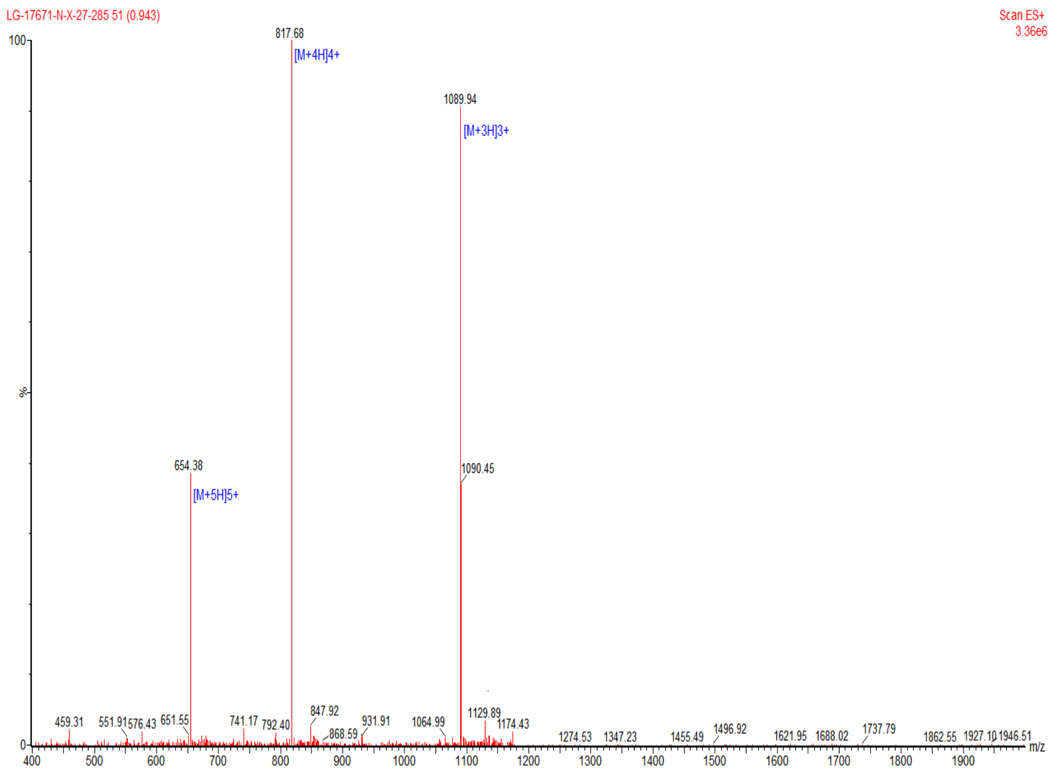

Figure S10. MS Analysis of N3-PEG2000-YTIWMPENPRPGTPCDIFTNSRGKRASNG.

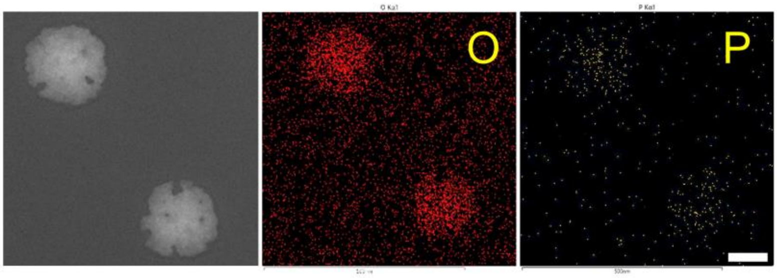

Figure S11. Elemental mapping of DK@R-PEG. Scale bar = 100 nm.

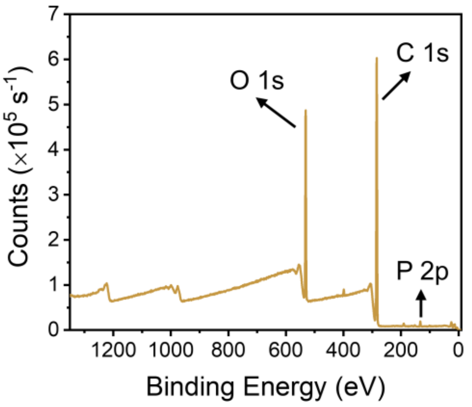

Figure S12. XPS measurement spectrum of DK@RA-PEG.

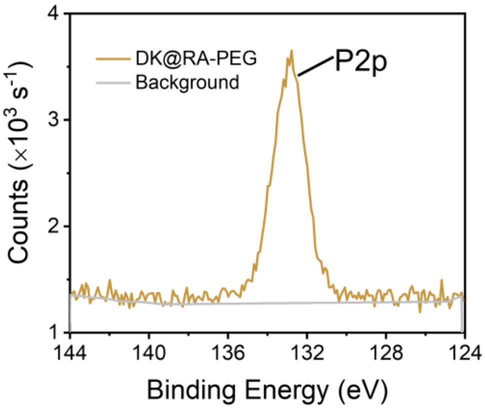

Figure S13. High resolution XPS spectra of P2p.

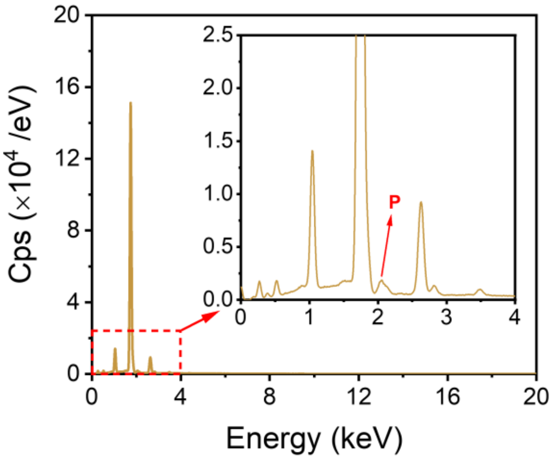

Figure S14. The EDS spectra of DK@RA-PEG.

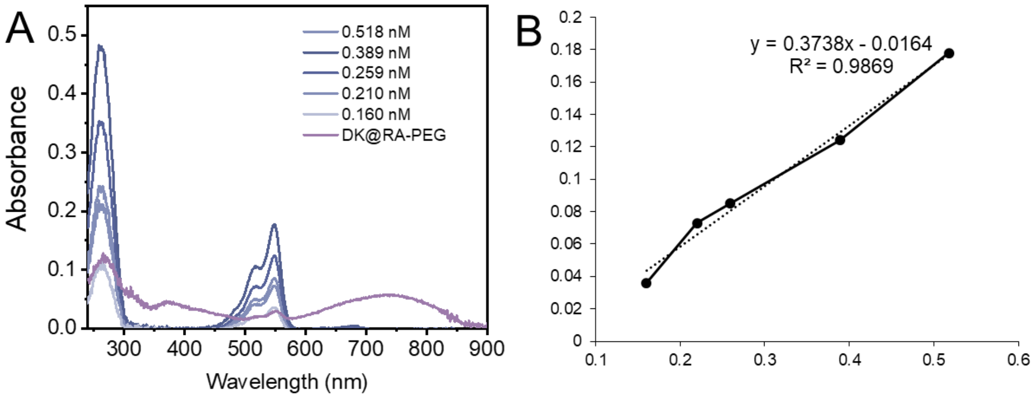

Figure S15. (A) Absorbance profiles of RVG-Cy3 (tested at 0.518, 0.389, 0.259, and 0.210 and 0.160 nM concentrations) compared to DK@RA-PEG. (B) Calibration curve of RVG-Cy3 concentration versus absorbance at λ = 550 nm. Linear regression equation: y = 0.3738x - 0.0164 (R^2 = 0.9869).

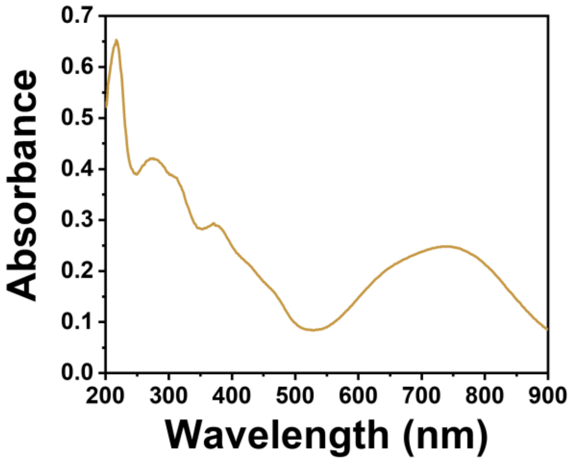

Figure S16. Absorbance of DK@RA-PEG in deionized water at the concentrations of 50 μM.

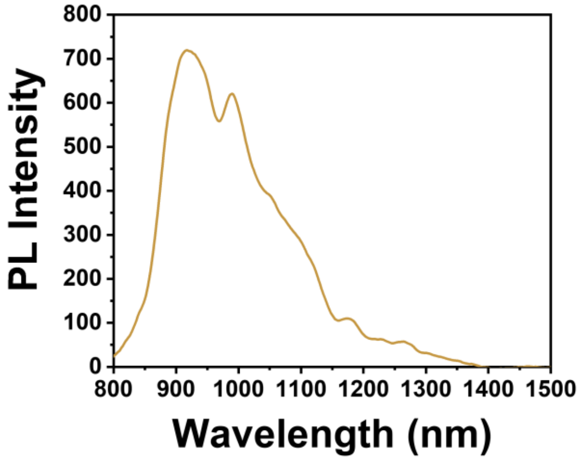

Figure S17. Fluorescent emission of DK@RA-PEG in deionized water at the concentrations of 10 μM.

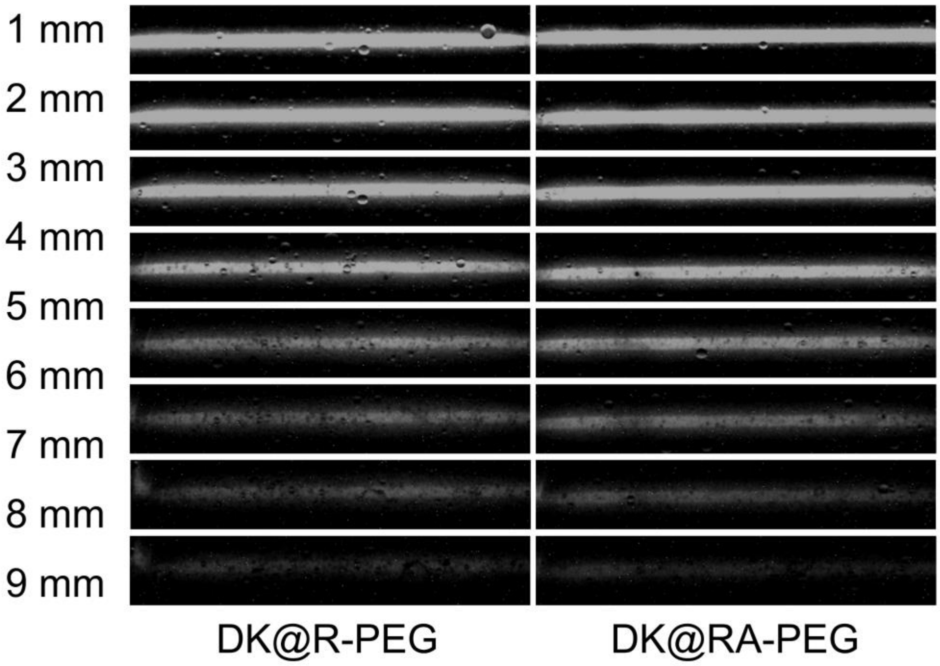

Figure S18. *In vitro* penetration depth in the NIR-II window (1000 nm Long-pass filter) using intralipid as tissue phantoms imitator.

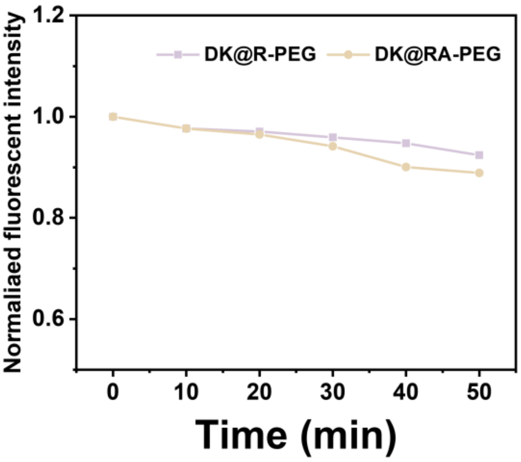

Figure S19. Photostability of DK@R-PEG and DK@RA-PEG in deionized water under continuous 808 nm irradiation (1.0 W/cm^2) and fluorescence intensity were recorded at predetermined time points (0, 5, 10, 20, 30, 40, and 50 min).

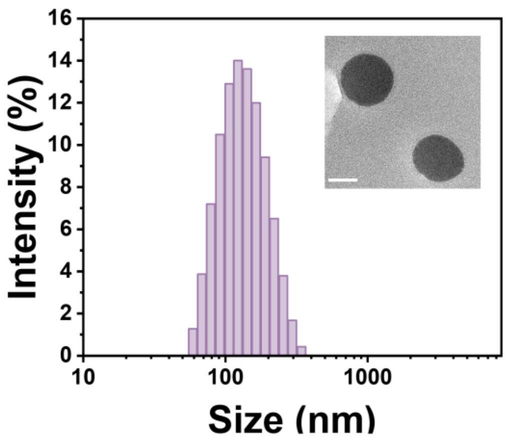

Figure S20. DLS size distribution and the representative TEM image of the **DK@R-PEG**. Scale bar: 100 nm.

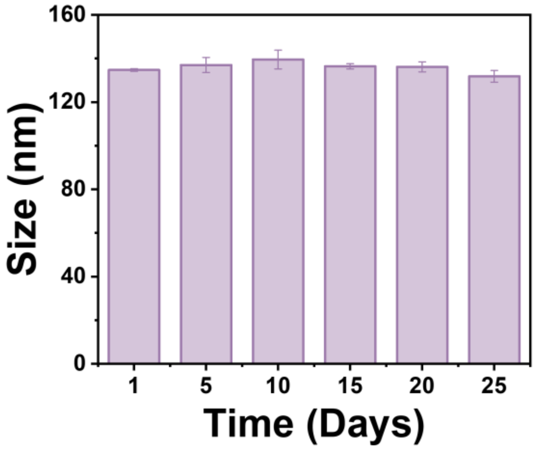

Figure S21. The size stability of **DK@R-PEG** (20 μM) in DMEM for 25 days.

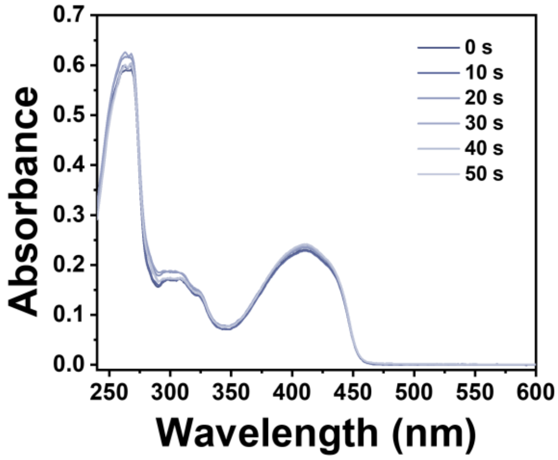

Figure S22. Absorption spectrum of DPBF solution under the conditions of 808 nm light irradiation. (0.5 W/cm^2).

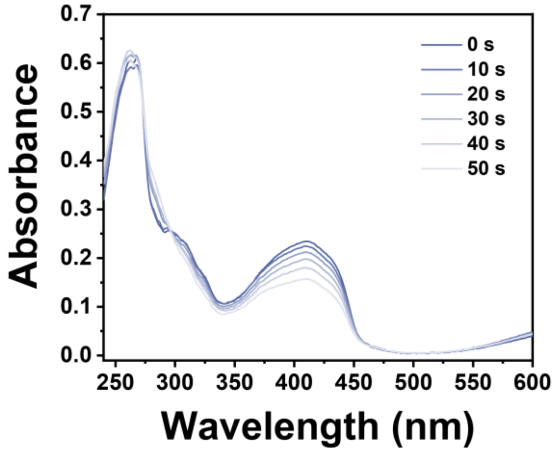

Figure S23. Absorption spectrum of DPBF solution containing 20 μM DK under 808 nm irradiation (0.5 W/cm^2).

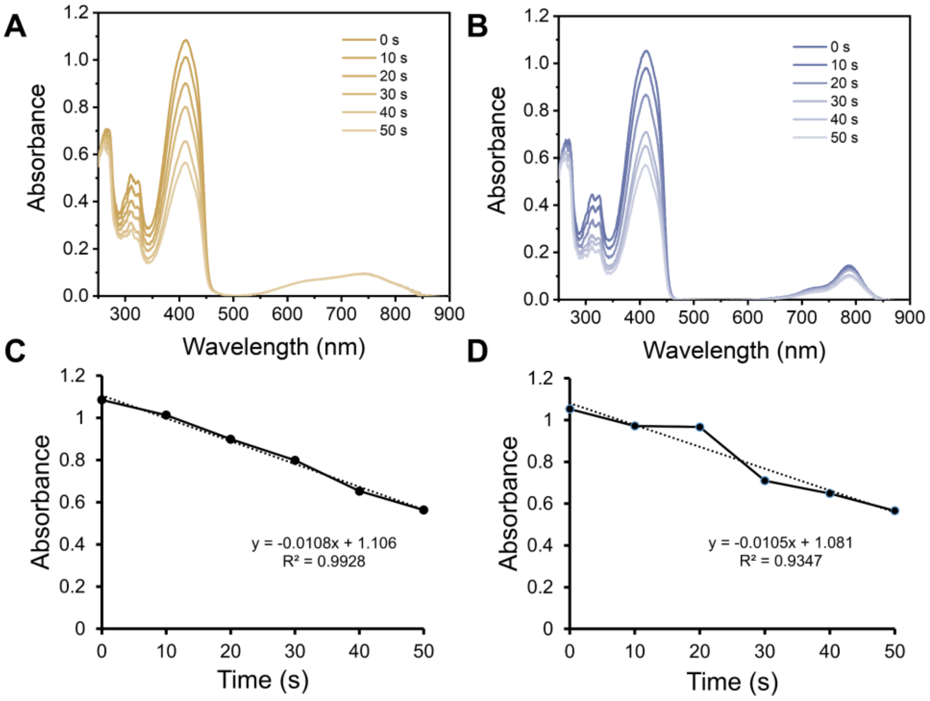

Figure S24. (**A**) Absorption spectrum of DK in DPBF solution under the conditions of 808 nm light irradiation (0.5 W/cm^2). (**B**) Absorption spectrum of ICG in DPBF solution under the conditions of 808 nm light irradiation (0.5 W/cm^2). (**C**) Calibration curve correlating the reduction in DPBF (DK) absorbance at $\lambda = 410$ nm with irradiation time under the conditions of 808 nm light irradiation (0.5 W/cm^2). First-order kinetic fitting: $y = -0.0108x + 1.106$ ($R^2 = 0.9928$). (**D**) Calibration curve correlating the reduction in DPBF (ICG) absorbance at $\lambda = 410$ nm with irradiation time under the conditions of 808 nm light irradiation (0.5 W/cm^2). First-order kinetic fitting: $y = -0.0105x + 1.081$ ($R^2 = 0.9347$).

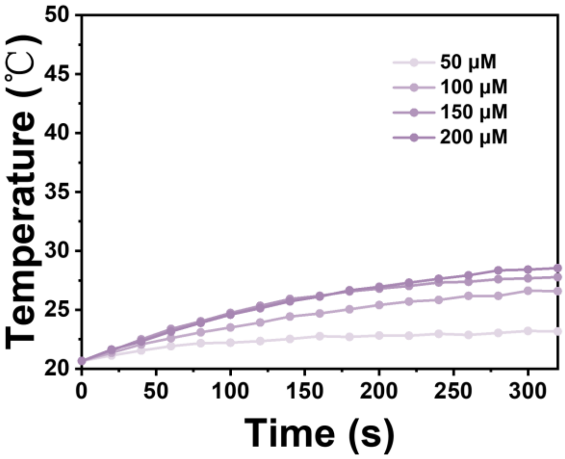

Figure S25. Temperature change curves of **DK@RA-PEG** (50, 100, 150 and 200 μg/ml) under the laser irradiation (808 nm, 0.5 W/cm^2) at various concentrations.

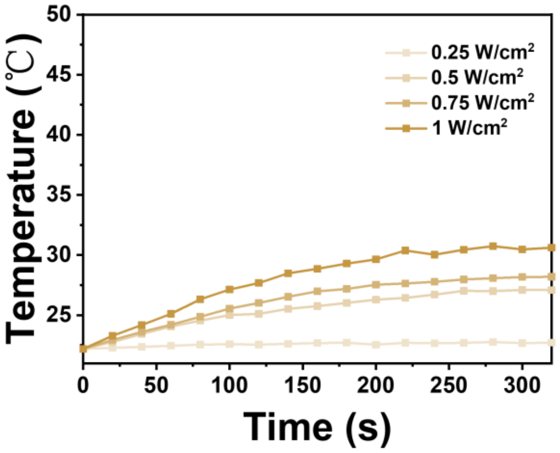

Figure S26. Temperature change curves of **DK@RA-PEG** (100 μg/ml) under the various 808 nm laser power irradiation (0.25, 0.5, 0.75 and 1 W/cm^2).

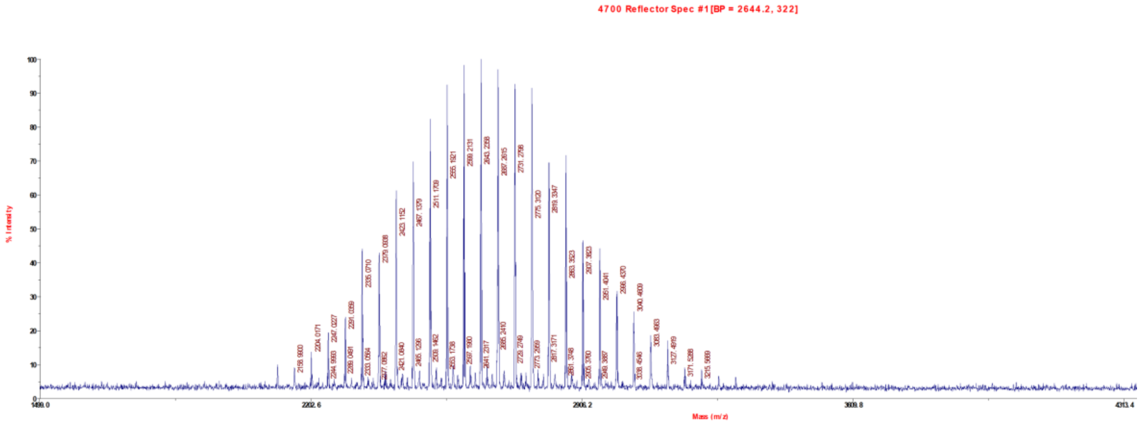

Figure S27. MALDI-TOF-MS spectrum of DK@PEG.

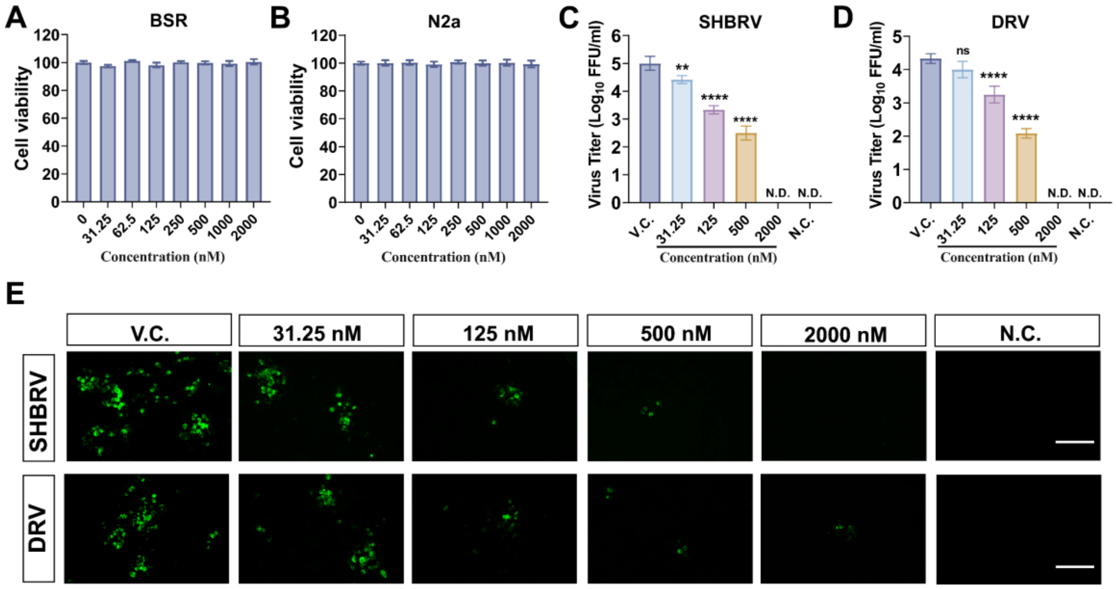

Figure S28. The antiviral activity of DK@RA-PEG on other RABV strains *in vitro*. (**A-B**) CCK-8 assays were used to determine the cytotoxicity of DK@RA-PEG to BSR cells(**A**) and N2a cells (**B**) (n = 5). (**C-D**) Virus titer of RABV-SHBRV (**C**) and RABV-DRV (**D**) after DK@RA-PEG-tread in N2a cells were examined by virus titration assay (n = 3). (**E**) Immunofluorescence assays of RABV-SHBRV or RABV-DRV infected N2a cells treated with different concentrations of DK@RA-PEG under 808 nm laser irradiation (0.5 W/cm^2) for 1 min, scale bar: 200 μm. Data are represented as mean ± SD. Statistical significance in (**C**) and (**D**) were calculated by one-way ANOVA with Tukey's multiple comparisons test. $^{**}P < 0.01$, $^{****}P < 0.0001$, N.D., not detected.

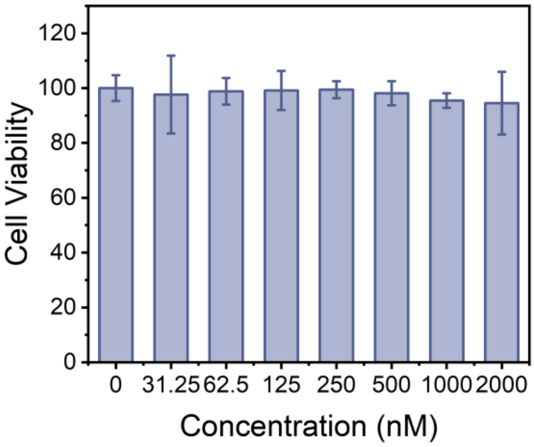

Figure S29. CCK-8 assays were used to determine the cytotoxicity of DK@RA-PEG to Bend.3 cells. (n = 5).

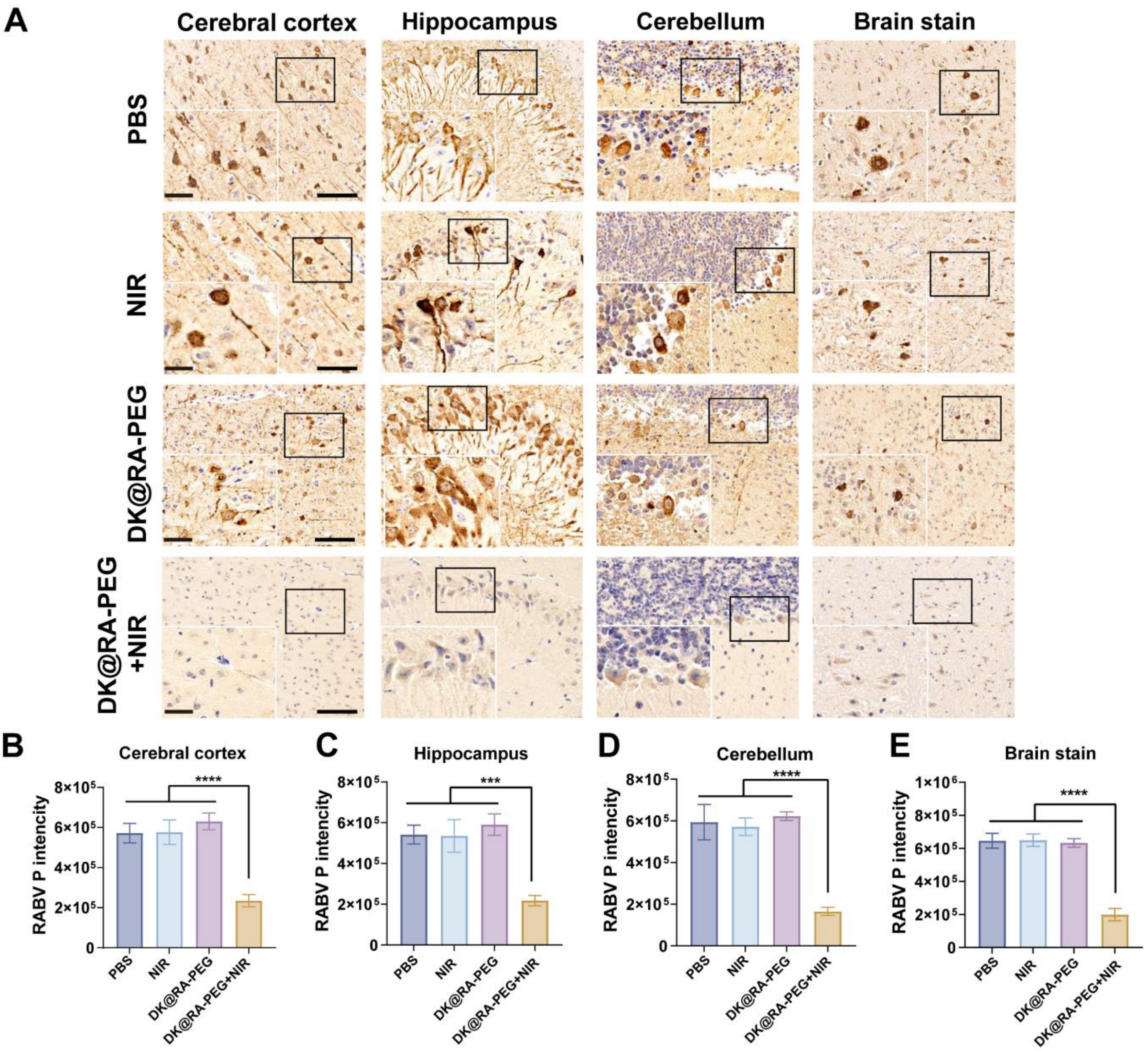

Figure S30. Immunohistochemistry analysis in the brain of representative mice from different experimental groups. (**A**)Immunohistochemistry (IHC) analysis in different brain areas (cerebral cortex, hippocampus, cerebellum, and brain stem) of mice under four different treatments. Scale bar, left: 50 μm, right: 200 μm. (**B-E**) RABV-P protein IHC intensity in the cerebral cortex (**B**), hippocampus (**C**), cerebellum (**D**), and brain stem (**E**) (n = 3). Data are represented as mean ± SD. Adjusted *P* values in (**B-E**) were calculated by one-way ANOVA with Tukey's multiple comparisons test. ****P* < 0.001, *****P* < 0.0001.

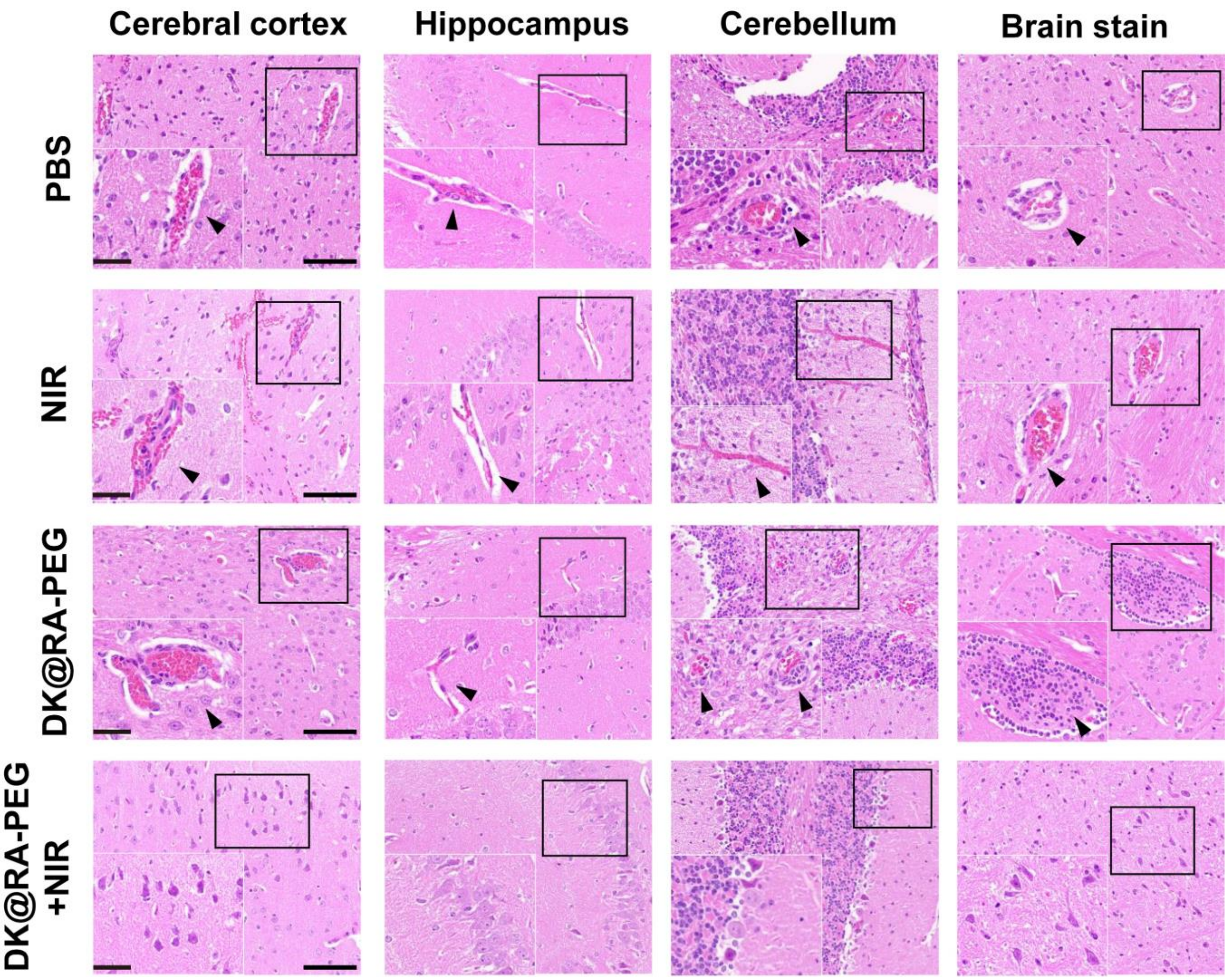

Figure S31. Histopathological analysis in the different brain areas by hematoxylin and eosin (H&E) staining of RABV infected mice (at 8 dpi) and surviving mice (at 21 dpi) treated of PDT. Scale bars: left: 50 μm, right: 200 μm.

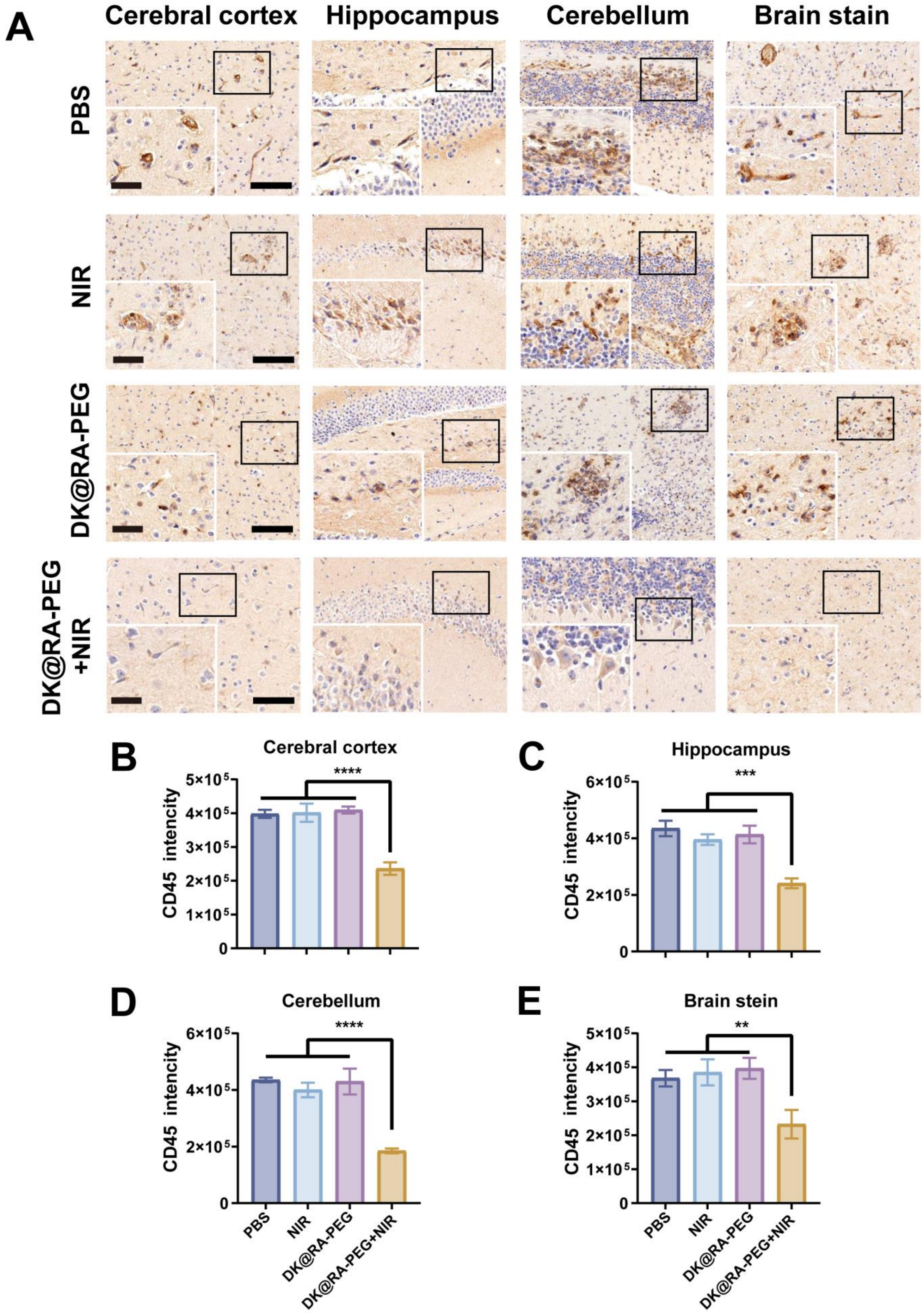
A
Cerebral cortex
Hippocampus
Cerebellum
Brain stain
PBS
NIR
DK@RA-PEG
DK@RA-PEG +NIR
B
Cerebral cortex
CD45 intencity
****
C
Hippocampus
***
D
Cerebellum
****
E
Brain stein
**
PBS
NIR
DK@RA-PEG
DK@RA-PEG+NIR

Figure S32. Neurological inflammation in RABV-infected mouse brains post-PDT. (**A**) Neurological inflammation was determined by quantifying $CD45^{+}$ cells in the different brain areas of RABV infected mice (at 8 dpi) and surviving mice (at 21 dpi) treated of PDT via IHC analysis. Scale bars, left: 50 μm, right: 200 μm. (**B-E**) CD45 protein IHC intensity in the cerebral cortex (**B**), hippocampus (**C**), cerebellum (**D**), and brain stem (**E**) (n = 3). Data are represented as mean ± SD. Adjusted *P* values in (**B-E**) were calculated by one-way ANOVA with Tukey's multiple comparisons test. $^{**}P < 0.01$, $^{***}P < 0.001$, $^{****}P < 0.0001$.

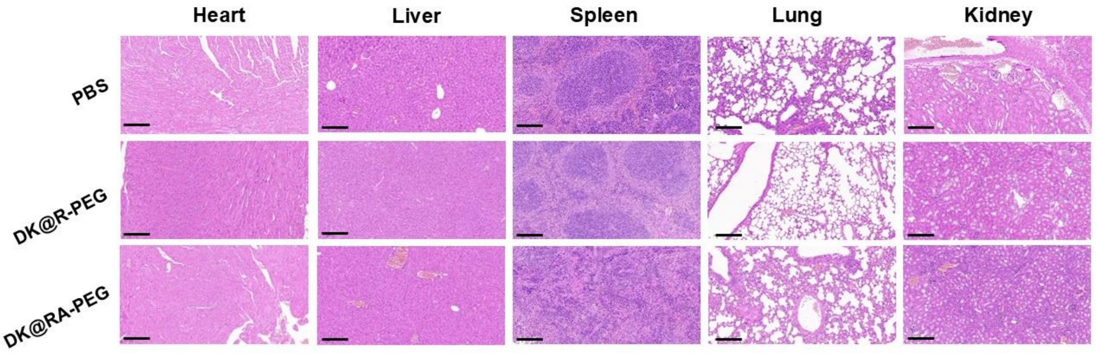

Figure S33. H&E staining of heart, liver, spleen, lung, and kidney in the PBS, DK@R-PEG, and DK@RA-PEG group. Scale bar: 100 μm.

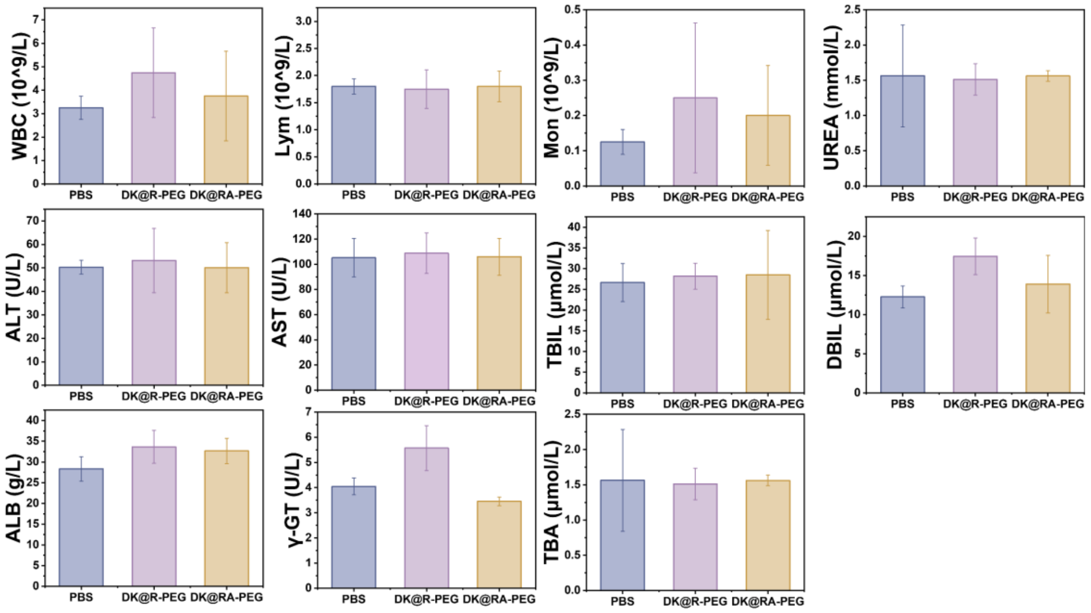

Figure S34. Hepatic and renal function and blood biochemical analysis post-injection. The terms were the following: WBC (White Blood Cell Count), Lym (Lymphocyte Percentage), MON (Monocyte Percentage), UREA (Urea), ALT (Alanine Aminotransferase), AST (Aspartate Aminotransferase), TBIL (Total Bilirubin), DBIL (Direct Bilirubin), ALB (Albumin), γ-GT (Gamma-Glutamyl Transferase), TBA (Total Bile Acids).

Table S1 Primers for qRT-PCR

Name	Sequences (5′-3′)
RABV vRNA-F	CTCCACAACGAGATGCTCAA
RABV vRNA-R	CATCCAACGGGAACAAGACT
RABV-N mRNA-F	CCTCGTCGTCAGAGTTGACA
RABV-N mRNA-R	GAGGAATTCTTCGGGAAAGG
Mouse β-actin-F	CACTGCCGCATCCTCTTCCTCCC
Mouse β-actin-R	CAATAGTGATGACCTGGCCGT

Table S2 The relative expression levels of RABV-N at each time point.

d	Spinal cord			Brain stem			cerebellum			cerebrum		
4	9.53E+00	5.57E+00	3.56E+00	7.57E-03	3.54E-02	1.73E-02	4.49E-02	9.21E-03	1.52E-02	8.76E-04	9.61E-05	7.46E-05
5	8.29E+00	9.70E+00	5.62E+00	1.93E+00	6.35E+00	4.44E+00	3.75E+00	3.92E+00	8.35E+00	4.10E-02	3.48E-02	7.75E-03
6	9.97E+00	7.79E+00	1.11E+01	4.51E+00	6.22E+00	5.88E+00	1.50E+01	2.14E+01	5.25E+00	1.51E+00	1.11E+00	1.20E+00
7	1.19E+01	1.23E+01	1.06E+01	7.75E+00	1.27E+01	6.50E+00	1.58E+01	2.12E+01	1.53E+01	3.19E+00	7.17E+00	1.02E+00
8	1.72E+01	1.14E+01	1.15E+01	1.49E+01	1.16E+01	1.34E+01	1.01E+01	9.61E+00	1.35E+01	1.31E+01	7.62E+00	1.34E+01
9	1.74E+01	2.45E+01	1.84E+01	2.66E+01	2.04E+01	1.61E+01	4.92E+00	2.61E+00	3.96E+00	1.28E+01	1.82E+01	2.18E+01

References

(1) Wang, Z. W.; Sarmento, L.; Wang, Y.; Li, X.-q.; Dhingra, V.; Tseggai, T.; Jiang, B.; Fu, Z. F. Attenuated rabies virus activates, while pathogenic rabies virus evades, the host innate immune responses in the central nervous system. *J. Virol.* **2005**, *79* (19), 12554-12565.

(2) a) Zhang, G., Fu, Z. F. Complete genome sequence of a street rabies virus from Mexico. *J. Virol.***2012.** b) Dietzschold, B.; Morimoto, K.; Hooper, D.; Smith, J.; Rupprecht, C.; Koprowski, H. Genotypic and phenotypic diversity of rabies virus variants involved in human rabies: implications for postexposure prophylaxis. *J. Hum. Virol.* **2000**, *3* (1), 50-57.

(3) Morimoto, K.; Patel, M.; Corisdeo, S.; Hooper, D. C.; Fu, Z. F.; Rupprecht, C. E.; Koprowski, H.; Dietzschold, B. Characterization of a unique variant of bat rabies virus responsible for newly emerging human cases in North America. *Proc. Natl. Acad. Sci. U. S. A.* **1996**, *93* (11), 5653-5658.

(4) Wang, C.; Lv, L.; Wu, Q.; Wang, Z.; Luo, Z.; Sui, B.; Zhou, M.; Fu, Z. F.; Zhao, L. The role of interferon regulatory factor 7 in the pathogenicity and immunogenicity of rabies virus in a mouse model. *J. Gen. Virol.* **2021**, *102* (10), 001665.

(5) Waldvogel, H. J.; Curtis, M. A.; Baer, K.; Rees, M. I.; Faull, R. L. Immunohistochemical staining of post-mortem adult human brain sections. *Nat. Protoc.* **2006**, *1* (6), 2719-2732.

(6) Zhang, T.; Chen, X.; Yuan, C.; Pang, X.; Shangguan, P.; Liu, Y.; Han, L.; Sun, J.; Lam, J. W.; Liu, Y. Near-Infrared Aggregation-Induced Emission Luminogens for In Vivo Theranostics of Alzheimer's Disease. *Angew. Chem. Int. Ed.* **2023**, *62* (2), e202211550.

(7) Chen, S.; Zhou, Y.; Chen, Y.; Gu, J. fastp: an ultra-fast all-in-one FASTQ preprocessor. *Bioinformatics* **2018**, *34* (17), i884-i890.

(8) Kim, D.; Langmead, B.; Salzberg, S. L. HISAT: a fast spliced aligner with low memory requirements. *Nat. Methods*, **2015**, *12* (4), 357-360.

(9) Roberts, A.; Trapnell, C.; Donaghey, J.; Rinn, J. L.; Pachter, L. Improving RNA-Seq expression estimates by correcting for fragment bias. *Genome Biol.* **2011**, *12*, 1-14.

(10) Anders, S.; Pyl, P. T.; Huber, W. HTSeq--a Python framework to work with high-throughput sequencing data. *Bioinformatics* **2015**, *31* (2), 166-169.

(11) Love, M. I.; Huber, W.; Anders, S. Moderated estimation of fold change and dispersion for RNA-seq data with DESeq2. *Genome Biol.* **2014**, *15*, 1-21.

(12) Yu, G.; Wang, L.-G.; Han, Y.; He, Q.-Y. clusterProfiler: an R package for comparing biological themes among gene clusters. *OMICS* **2012**, *16* (5), 284-287.
